# Induced production of specialized steroids by transcriptional reprogramming in *Petunia hybrida*

**DOI:** 10.1093/pnasnexus/pgad326

**Published:** 2023-10-31

**Authors:** Tsubasa Shoji, Satoko Sugawara, Tetsuya Mori, Makoto Kobayashi, Miyako Kusano, Kazuki Saito

**Affiliations:** RIKEN Center for Sustainable Resource Science, Yokohama, Kanagawa 230-0045, Japan; Institute of Natural Medicine, University of Toyama, Toyama, Toyama 930-0194, Japan; RIKEN Center for Sustainable Resource Science, Yokohama, Kanagawa 230-0045, Japan; RIKEN Center for Sustainable Resource Science, Yokohama, Kanagawa 230-0045, Japan; RIKEN Center for Sustainable Resource Science, Yokohama, Kanagawa 230-0045, Japan; RIKEN Center for Sustainable Resource Science, Yokohama, Kanagawa 230-0045, Japan; Graduate School of Life and Environmental Science, University of Tsukuba, Tsukuba, Ibaraki 305-8572, Japan; Tsukuba-Plant Innovation Research Center (T-PIRC), University of Tsukuba, Tsukuba, Ibaraki 305-8572, Japan; RIKEN Center for Sustainable Resource Science, Yokohama, Kanagawa 230-0045, Japan

**Keywords:** gene cluster, petunia, specialized metabolite, steroid, transcription factor

## Abstract

Plants produce specialized metabolites with defensive properties that are often synthesized through the coordinated regulation of metabolic genes by transcription factors in various biological contexts. In this study, we investigated the regulatory function of the transcription factor PhERF1 from petunia (*Petunia hybrida*), which belongs to a small group of ETHYLENE RESPONSE FACTOR (ERF) family members that regulate the biosynthesis of bioactive alkaloids and terpenoids in various plant lineages. We examined the effects of transiently overexpressing *PhERF1* in petunia leaves on the transcriptome and metabolome, demonstrating the production of a class of specialized steroids, petuniolides, and petuniasterones in these leaves. We also observed the activation of many metabolic genes, including those involved in sterol biosynthesis, as well as clustered genes that encode new metabolic enzymes, such as cytochrome P450 oxidoreductases, 2-oxoglutarate-dependent dioxygenases, and BAHD acyltransferases. Furthermore, we determined that PhERF1 transcriptionally induces downstream metabolic genes by recognizing specific *cis*-regulatory elements in their promoters. This study highlights the potential of evolutionarily conserved transcriptional regulators to induce the production of specialized products through transcriptional reprogramming.

**Significance Statement**

Plants produce many specialized metabolites that have been harnessed as valuable compounds in our daily lives, and they are increasingly recognized as sustainable alternatives to synthetic chemicals. In this study, we show that the overexpression of a transcriptional regulator from the ERF family in petunia induces the production of defense-related steroids through the activation of sterol biosynthesis and mostly clustered metabolic genes. Our findings highlight the potential use of evolutionarily conserved factors as genetic tools to effectively elicit the production of bioactive natural products by reprogramming transcription, without requiring prior knowledge of downstream metabolic pathways.

## Introduction

Plants have a tremendous ability to produce and accumulate a wide variety of structurally complex and diverse specialized metabolites, also known as secondary metabolites, including bioactive alkaloids and terpenoids (1). These compounds are generally specific to species or groups of taxonomically related species and are present in limited quantities within plant tissues. They often confer adaptive advantages to the producing plants, especially in fluctuating environmental conditions. Humans have long exploited plant-derived natural products as medicines, dyes, perfumes, and industrial materials. It is important to develop plant and other biological resources to ensure a stable supply of these valuable chemicals.

Recent developments in molecular and genomics approaches have greatly facilitated the discovery and functional characterization of genes encoding metabolic enzymes involved in the biosynthesis of specialized metabolites in plants (2). In certain instances, such as with morphine, tropane alkaloids, and monoterpenoid indole alkaloids, an entire series of metabolic genes have been defined and exploited to reconstitute the biosynthesis pathway in heterogeneous microorganisms (3–5). However, to employ this synthetic approach successfully, it is critical to identify many metabolic genes that form an entire biosynthesis pathway. Conventional gene discovery, which largely relies on structural homology and expression profiles, has limitations, and new approaches are required.

To ensure metabolic flow through multiple enzymatic steps and rapid metabolic adjustment in response to developmental and environmental stimuli, the expression of metabolic genes in certain pathways is often coordinated by transcription factors (6, 7). These factors positively and/or negatively regulate transcription of downstream target genes by specifically recognizing *cis*-regulatory elements in their promoters. Natural variation in and genetic manipulation of genes encoding such transcriptional regulators often leads to substantial metabolic alterations (8, 9), as exemplified by anthocyanin-based color diversification in flowers and fruits (10–12) and the elimination of toxic substances in major crops (13–15). In contrast to microbial production mentioned above, this metabolic engineering approach, which relies on transcriptional regulation, does not necessarily require much knowledge of the downstream metabolic steps.

The plant-specific APETALA2/ETHYLENE RESPONSE FACTOR (AP2/ERF) family is one of the largest transcription factor families, with over 100 members in each species (16). A small group of ERFs from clade II of group IXa (16, 17) are known transcriptional regulators in diverse specialized pathways from different plant lineages (18). These ERFs include CrORCAs (Octadecanoid-derivative-responsive Catharanthus AP2-domain), which regulate monoterpenoid indole alkaloids in Madagascar periwinkle (*Catharanthus roseus*) (19, 20); the homologous pair NtERF189 and NtERF199, which regulate nicotine biosynthesis in tobacco (*Nicotiana tabacum*) (21, 22); SlJRE4 (jasmonate-responsive ERF 4), which regulates steroidal glycoalkaloids in tomato (*Solanum lycopersicum*) (23, 24); and AaORA (Octadecanoid-responsive AP2), which regulates artemisinin in sweet wormwood (*Artemisia annua*) (25). The expression of the genes encoding the reported ERFs in this group and their downstream pathways are commonly induced by jasmonates (18), a class of fatty acid–derived phytohormones that play central roles in plant defense against herbivores and pathogens (26). Most *ERF* genes are found in tandem clusters in plant genomes (18). Clusters of 10, 5, and 5 *ERF* genes have been reported in the tobacco (27), tomato (24), and *C. roseus* (20) genomes, respectively. As genome sequences have become available in many plants, related clustered *ERF* genes have been discovered in many eudicots (18). It remains unclear whether these newly identified *ERF* genes also play regulatory roles in defense metabolism or other pathways in their respective species.

Petunia (*Petunia hybrida*) is an ornamental flowering species in the family Solanaceae, which produces and accumulates specialized compounds, such as steroids (28) and acylsugars (29), with protective properties. In this study, we identified a group of clustered genes in the petunia genome, named *PhERF* genes, which are related to *NtERF189* and *SlJRE4*. We investigated the *in planta* function of PhERF1 as a transcriptional regulator in the biosynthesis of specialized steroids, such as petuniolides and petuniasterones, by analyzing the effects of transient *PhERF1* overexpression in petunia leaves on the transcriptome and metabolome. Importantly, we had no a priori knowledge of the pathway under the control of this ERF, but we elucidated the entire biosynthetic pathway relying on this ERF using a multiomics approach. Our findings provide another example of ERFs regulating defense metabolism in plants and demonstrate a promising strategy for enhancing the production of bioactive specialized metabolites by reprogramming transcription networks using evolutionarily conserved transcription factors.

## Results

### Identification of petunia ERF transcription factors related to regulators of nicotine and steroidal glycoalkaloid biosynthesis

**Fig. 1.**
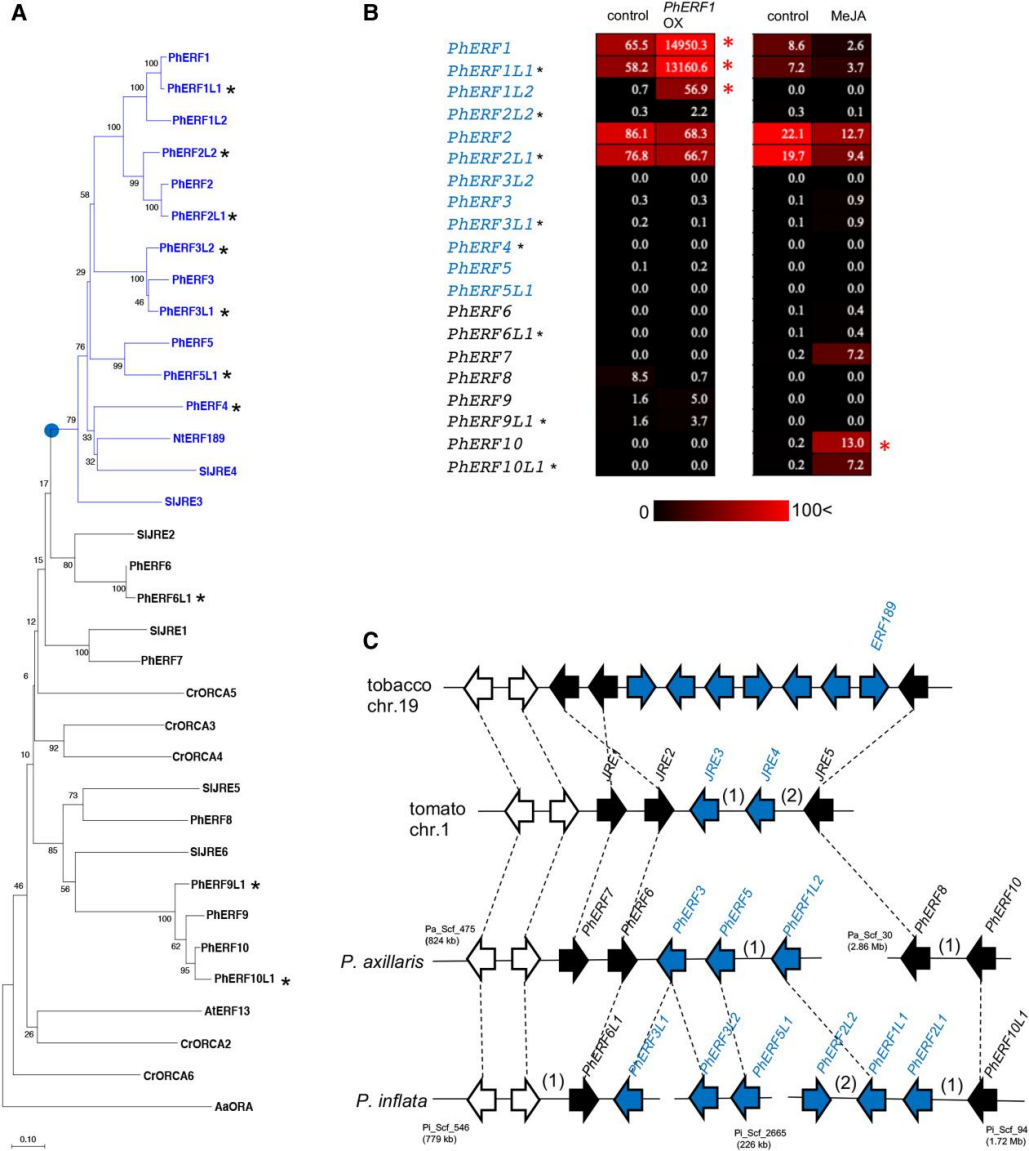
Identification of petunia ERF transcription factors related to tomato JREs and tobacco ERF189. A) Phylogenetic tree of ERFs from various plant species. PhERFs from petunia (Ph; *P. hybrida*) predicted from the *P. axillaris* (v.1.6.2) and *P. inflata* (v. 1.0.1) genomes; SlJRE1 (Solyc01g090300), SlJRE2 (Solyc01g090310), SlJRE3 (Solyc01g090320), SlJRE4 (Solyc01g090340), SlJRE5 (Solyc01g090370), and SlJRE6 (Solyc05g050790) from tomato (Sl; *S. lycopersicum*, ITAG release 2.40); NtERF189 (GenBank; AB827951) from tobacco (Nt; *N. tabacum*); CrORCA2 (GenBank; AJ238740), CrORCA3 (GenBank; AJ251250), CrORCA4 (GenBank; MT414982), CrORCA5 (GenBank; MT414981), and CrORCA6 (GenBank; MT414983) from Madagascar periwinkle (Cr; *C. roseus*); AaORA (GenBank; JQ797708) from sweet wormwood (Aa; *A. annua*); and AtERF13 (At2g44840) from Arabidopsis (At; *A. thaliana*) are included. ERFs, which belong to one clade with a branch point marked with a circle, are less conserved among tobacco, tomato, and petunia (see text). The percentage support from 1,050 bootstrap replicates is indicated at branch nodes. The scale bar indicates the number of amino acid substitutions per site. PhERFs predicted from the *P. inflata* genome are marked with asterisks. B) Heatmap illustration of *PhERF* expression levels in petunia leaves. Normalized expression values obtained by RNA-seq analysis in petunia leaves transiently overexpressing *PhERF1* (left) or treated with MeJA (right). Genes with significant changes in expression upon *PhERF1* overexpression or MeJA treatment (log_2_-transformed FC ≥ 1 or ≤ −1 and adjusted *P*-value < 0.05) are marked with asterisks. PhERFs predicted from the *P. inflata* genome are marked with asterisks. Details are available in Table S1. C) Clusters of *ERF* genes in tobacco, tomato, and petunia genomes. Petunia has a homologous pair of presumptive clusters, which are represented with five scaffolds from the *P. axillaris* and *P. inflata* genomes. Positions and orientations of *ERF* genes are shown as arrows. White arrows represent non-*ERF* genes, which are present near the clusters and conserved among species. Numbers of non-ERF genes present between the arrowheads are shown in parentheses. Homologous genes are linked with dashed lines.

The genome of the garden petunia (*P. hybrida*, also called *Petunia×atkinsiana*) comprises the parental genomes of *Petunia axillaris* and *Petunia inflata* (30). Using a BLASTP search using tomato ERFs as queries, we retrieved a group of 20 related ERF-type transcription factors predicted in the *P. axillaris* (v1.6.2) and *P. inflata* (v1.0.1) genomes that are related to NtERF189 (GenBank: AB827951.1) from tobacco and SlJRE4 (Solyc01g090340) from tomato. We named these 20 PhERFs (using the prefix “Ph” to denote *P. hybrida*) based on their membership to 10 homologous subgroups (Fig. 1A, Table S1). Accordingly, we reconstructed a phylogenetic tree using the 20 PhERFs, 6 SlJREs (24), NtERF189, and AtERF13 (At2g44840) from Arabidopsis (*Arabidopsis thaliana*) (Fig. 1A). In the tree, 12 PhERFs (PhERF1–PhERF5 and their 7 relatives) form a single clade with NtERF189, SlJRE3, and SlJRE4 (Fig. 1A).

We examined *PhERF* expression levels in petunia leaves by transcriptome deep sequencing (RNA-seq) and plotted the data as heatmaps (Fig. 1B; Table S1). *PhERF1*, *PhERF2*, and their homoeologs *PhERF1L1* and *PhERF2L1* showed high expression levels in leaves, whereas the other members had limited or no expression (Fig. 1B; Table S1). To investigate the function of PhERF1, we transiently overexpressed it in petunia leaves using *Agrobacterium*-mediated transformation with a binary vector containing the full-length coding region of *PhERF1* under the control of the constitutive cauliflower mosaic virus (CaMV) 35S promoter and conducted an RNA-seq analysis (Data Sets S1 and S2). We detected much higher transcript levels for *PhERF1* and its close homologs, *PhERF1L1* and *PhERF1L2*, but not for other *PhERF* genes (Fig. 1B; Table S1), indicating that PhERF1 does not regulate *PhERF* genes in general. Please note the possibility of short-read misalignment for *PhERF1* with its closely related counterparts. Furthermore, we evaluated the response of petunia plants to methyl jasmonate (MeJA) exposure by RNA-seq analysis (Data Sets S3 and S4). *PhERF10* was significantly induced by MeJA, and its expression was predominant in MeJA-treated leaves, whereas MeJA did not significantly affect the expression of the other *PhERF* genes (Fig. 1B; Table S1).

The *PhERF* genes appeared to be present in the petunia genome as a pair of homologous clusters, consisting of 15 *PhERF* genes represented by 2 scaffolds in the *P. axillaris* genome and 3 scaffolds in the *P. inflata* genome (Fig. 1C). *PhERF1*, *PhERF2*, *PhERF4*, *PhERF9*, and *PhERF9L1* were not in these clusters. We observed partial synteny and similar gene arrangements among the *ERF* gene clusters in the petunia, tobacco, and tomato genomes, likely reflecting their common ancestry and diversification (Fig. 1C). Since PhERF1 belongs to the same clade as NtERF189 and SlJRE4 (Fig. 1A) and *PhERF1* is highly expressed in petunia leaves (Fig. 1B), we chose this gene for characterization.

### PhERF1 up-regulates metabolic genes involved in steroid biosynthesis

To explore the downstream genes regulated by PhERF1, we identified differentially expressed genes (DEGs) (Tables S2 and S3) in our RNA-seq data sets (Data Sets S1 and S2) that were up-regulated (log_2_-transformed fold-change [FC] > 1) or down-regulated (log_2_-transformed FC < −1) by *PhERF1* overexpression. Gene Ontology (GO) analysis revealed an enrichment for terms related to sterol or steroid metabolism in the up-regulated DEGs (Table S4).

**Fig. 2.**
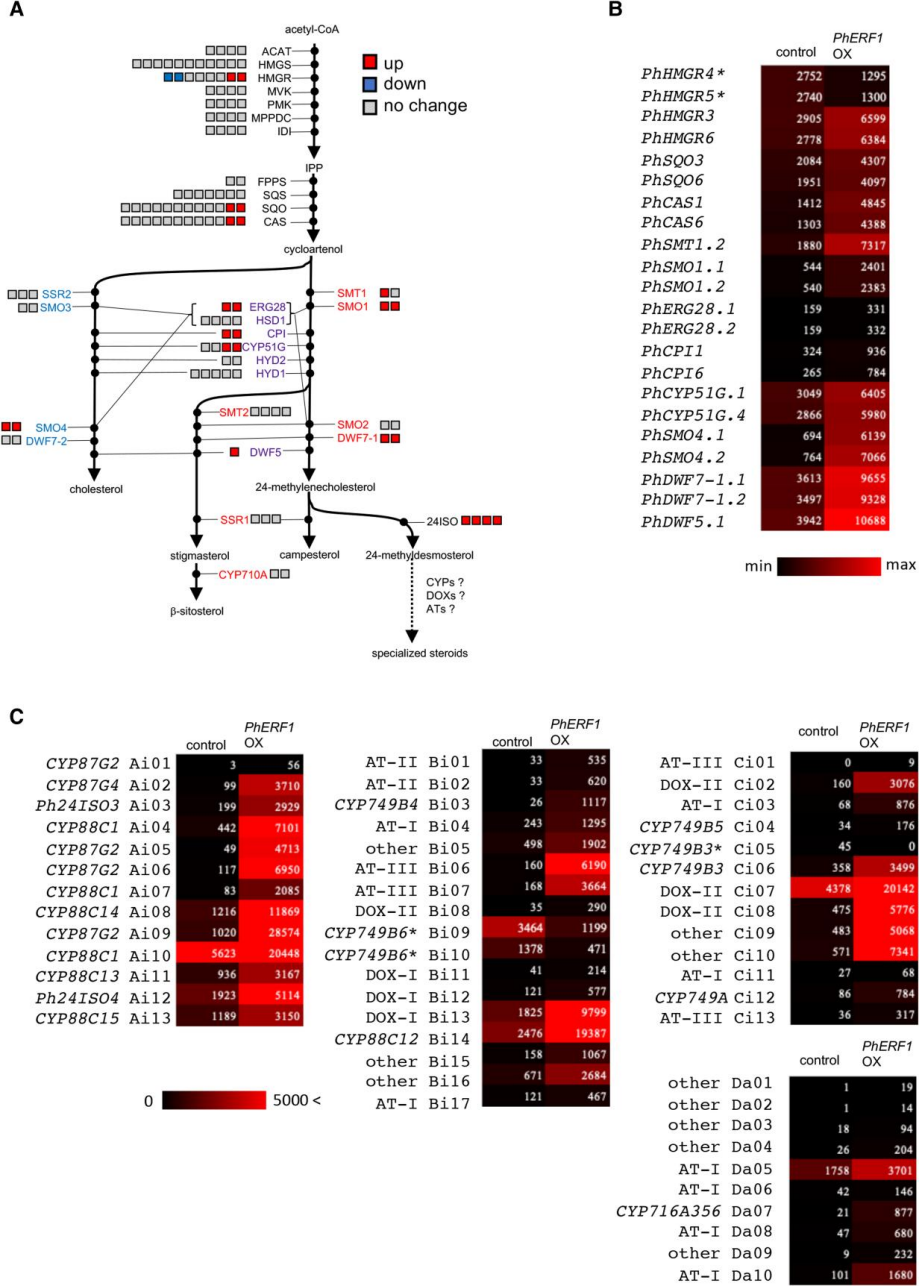
PhERF1 regulates newly predicted genes involved in sterol biosynthesis. A) Diagram of sterol biosynthesis and related pathways. Petunia biosynthesis genes involved in the pathways (Table S6) were predicted based on similarities to their tomato counterparts (Table S5). The genes are represented as boxes placed beside enzyme abbreviations (see Table S5); red and blue boxes represent genes up-regulated and down-regulated by PhERF1, respectively. Downstream of cycloartenol, cholesterol-specific genes are shown in blue; phytosterol-specific genes are shown in red, and genes involved in both pathways are in purple. *Petunia*-specific steroids, such as petuniolides and petuniasterones, are presumed to be produced through a downstream branch that starts with a reaction mediated by sterol 24ISO. B) and C) Heatmap visualization of the expression levels of PhERF1-regulated genes shown in A and genes present in clusters Ai, Bi, Ci, and Da (see Fig. 3). *24ISO* genes are included in C. Please refer to Table S5 or Fig. 3 for gene names and labels. Normalized expression values obtained in leaves transiently overexpressing *PhERF1* are shown. PhERF1-down-regulated genes are marked with asterisks.

To better annotate the relevant biosynthetic pathways in petunia, we performed a BLASTP search using a set of tomato proteins (Table S5) as queries to identify petunia proteins involved in sterol metabolism. We annotated 117 genes from petunia involved in sterol metabolism and related pathways from acetyl-CoA to cholesterol, phytosterols, and specialized steroids (Table S6). The common upstream pathway splits into multiple downstream branches after the common intermediate cycloartenol (Fig. 2A). Based on phylogenetic analysis with their tomato counterparts (31), we defined petunia metabolic proteins specific to each branch and those shared between branches (Fig. 2A; Fig. S1). We noticed that enzymes mediating most of the steps in the upstream part of the pathway before cycloartenol are encoded by multiple pairs of genes (Fig. 2A; Table S6), possibly hinting at complex regulation to meet metabolic demands for sustaining metabolite flow to multiple downstream branches.

We determined that PhERF1 induces one pair of homoeolog genes encoding either hydroxymethylglutaryl-CoA reductase (HMGR), squalene monooxygenase (SQO), or cycloartenol synthase (CAS) while down-regulating another pair of *HMGR* genes (Fig. 2A and B; Tables S2 and S3). In the downstream portion of the pathway, both pairs of homoeolog genes encoding sterol Δ24-isomerase (24ISO) were up-regulated by PhERF1 (Fig. 2A and C, Table S2). In several species of the family Solanaceae, 24ISO was identified as a key enzyme that catalyzes the conversion of 24-methylenecholesterol to 24-methyldesmosterol, which is a precursor of certain specialized steroids, such as withanolides in *Withania* species (32) and physalins in *Physalis* species (33). Thus, the PhERF1-mediated up-regulation of *24ISO*s implies the involvement of this ERF factor in transcriptional activation of a biosynthetic pathway leading to 24-methyldesmosterol-derived steroidal compounds in petunia like in other Solanaceae species. In line with this idea, with the exception of *STEROL METHYL OXIDASE 4* (*SMO4*) genes thought to be specific for cholesterol biosynthesis (Fig. 2A) (31), PhERF1 up-regulated the expression of metabolic genes encoding the enzymes sterol methyltransferase 1 (SMT1), sterol methyl oxidase 1 (SMO1), ERG28-like protein (ERG28), cyclopropylsterol isomerase (CPI), sterol C14-demethylase (CYP51G), sterol C-5 desaturase/Dwarf 7-1 (DWF7-1), and sterol reductase (also known as DWF5). Notably, all these enzymes, whether specific to one branch or shared between branches, belong to the biosynthetic route contributing to specialized steroids (Fig. 2A and B; Table S2) (31).

**Fig. 3.**
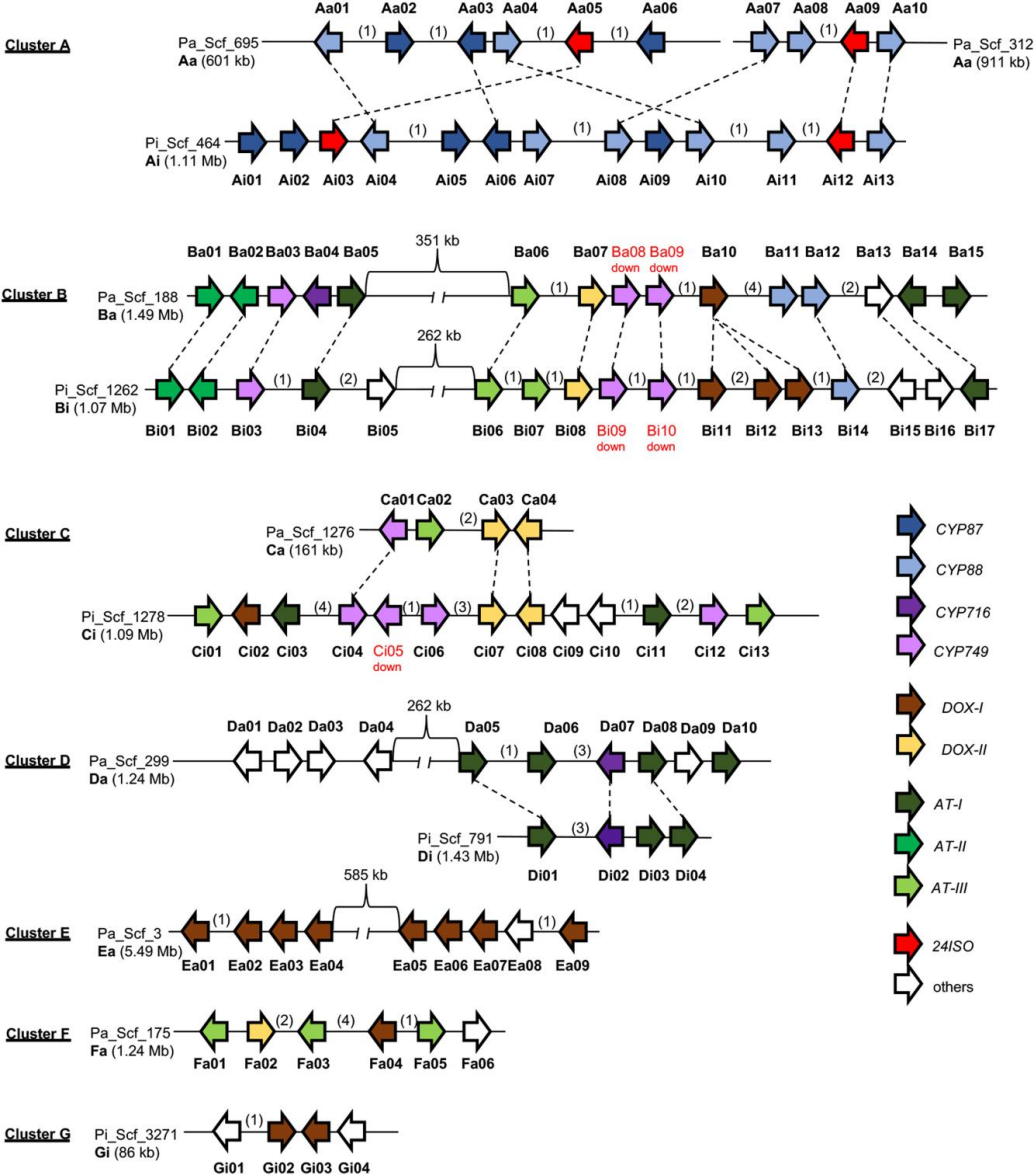
Multiple clusters of PhERF1-regulated genes exist in *Petunia* genomes. PhERF1-regulated genes (≥4) were considered to form clusters in the genomes when present in tandem with ≤4 nonrelevant genes between any two genes. Positions and orientations of PhERF1-regulated genes (arrows; 100 up-regulated and 5 down-regulated DEGs, whose details are in Tables S2 and S3, respectively) are represented on scaffolds (Scf) from the *P. axillaris* (v.1.6.2) and *P. inflata* (v.1.0.1) genomes with labels indicating clusters and positions (numbered from left to right on each scaffold). Five genes down-regulated by PhERF1 are shown with red labels. Numbers of unrelated genes present between the arrows are shown in the brackets. Arrow colors reflect protein classification: AT, BAHD-type acyltransferase; CYP, cytochrome P450; DOX, 2-oxoglutarate-dependent dioxygenase, 24ISO; sterol Δ24-isomerase. Four groups of clusters, A, B, C, and D, each of which is represented by a homologous pair of clusters from *P. axillaris* and *P. inflata* (respectively denoted a and i) were presumed in petunia, whereas clusters E, F, and G are each present in only one of the two genomes. Genes closely related between *P. axillaris* and *P. inflata* sequences (see Figs. S2–S4) are linked with dashed lines.

We detected 100 up-regulated DEGs, including all 4 *24ISO* genes, and 5 down-regulated DEGs, which together formed 7 different types of 11 presumptive clusters, including 8 clusters containing 4 homoeologous pairs (Fig. 3). Most of the clustered DEGs and 25 homologous DEGs not in clusters encoded metabolic enzymes not assigned to specific pathways, belonging to the cytochrome P450 (CYP) (34), 2-oxoglutarate-dependent dioxygenase (DOX) (35), or BAHD acyltransferase (AT) (36) families (Table S2). We reconstructed phylogenetic trees using these CYPs, DOXs, and ATs from petunia and their homologs from petunia and tomato, which allowed us to classify each member into a few subgroups of each family (Figs. S2–S4). Related genes were usually located in close proximity in the petunia genome, suggesting cluster formation through tandem gene duplications (Fig. 3).

### Induction of metabolic genes by PhERF1 and related proteins

**Fig. 4.**
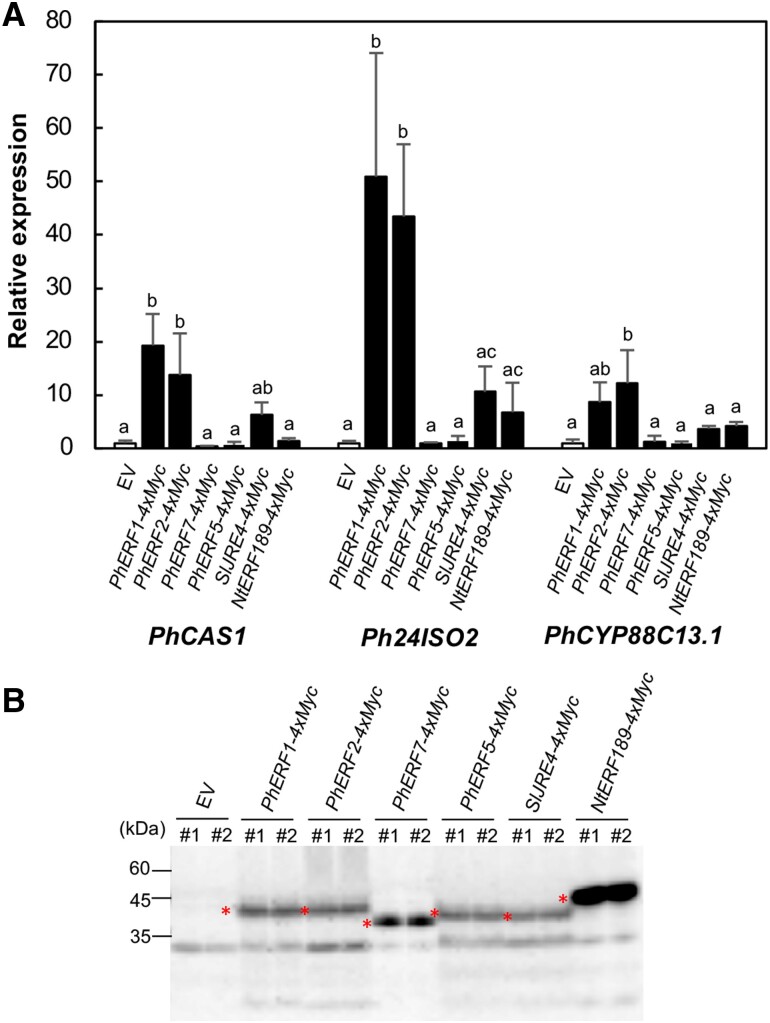
Transient overexpression of *PhERF1* and related *ERF* genes selectively induces metabolic genes in petunia leaves. *PhERF1* (Peaxi162Scf00672g00031), *PhERF2* (Peaxi162Scf01094g00002), *PhERF5* (Peaxi162Scf00475g00077), *PhERF7* (Peaxi162Scf00475g00053), *SlJRE4* (Solyc01g090340), and *NtERF189* (GenBank; AB827951) cloned in-frame and upstream of the sequence encoding a 4× Myc tag in pGWB417 (37) were individually transiently overexpressed in petunia leaves under the control of the CaMV 35S promoter. The pBI121 vector was used as the EV control. A) Relative expression levels of *PhCAS1* (Peaxi162Sch00263g00924), *Ph24ISO2* (Peaxi162Sch00695g00218), and *PhCYP88C13* (Peaxi162Sch00312g00220). Transcript levels were determined by RT-qPCR analysis and normalized to the abundance of the fusion proteins determined in B. The values in the controls (white bars) were set to 1. Values are means ± SD for biological replicates. Different lowercase letters indicate significant differences at *P* < 0.05, as determined by one-way ANOVA followed by a Tukey–Kramer test. B) Abundance of Myc-tagged ERF fusion proteins, as determined by immunoblot analysis with an anti-Myc antibody. Major detected bands are indicated with asterisks. Intensities of the bands were measured. Two biological replicates (#1 and #2) were analyzed.

We investigated the functional similarities and differences between PhERF1 and related proteins as transcriptional regulators. To this end, we delivered constructs encoding each full-length ERF protein fused to a 4× Myc tag at the C-terminal end via *Agrobacterium*-mediated infiltration of petunia leaves. To assess the transactivation of downstream genes by each introduced PhERF, we measured the expression levels of three PhERF1-regulated metabolic genes, *PhCAS1*, *Ph24ISO2*, and *PhCYP88C13.1*, by reverse transcription-quantitative PCR (RT-qPCR) analysis (Fig. 4A). As transcriptional output depends on the abundance of the transcription factor, we estimated the relative accumulation of each Myc-tagged PhERF in the leaf extracts by immunoblot analysis (Fig. 4B) and normalized transcript levels by the measured protein abundance. We observed that the expression of *PhCAS1*, *Ph24ISO2*, and *PhCYP88C13.1* was significantly induced, to similar degrees, upon overexpression of *PhERF1* or *PhERF2*, indicating that these two PhERFs have comparable transcriptional activation potential (Fig. 4A). In contrast, overexpression of *PhERF5* or *PhERF7* did not significantly affect the transcript levels of the examined metabolic genes compared with the empty vector (EV) controls (Fig. 4A). We also determined that SlJRE4 and NtERF189 significantly induce the expression of *PhCAS1* (in the case of SlJRE4) and *Ph24ISO2* (in the case of SlJRE4 and NtERF189), by 6.2- to 11-fold (Fig. 4A). These results indicate that the ERFs from tomato and tobacco have transactivation activities in petunia, but their activities are weaker than those of PhERF1 and PhERF2.

### PhERF1-mediated transcriptional regulation of metabolic genes through promoter regions

**Fig. 5.**
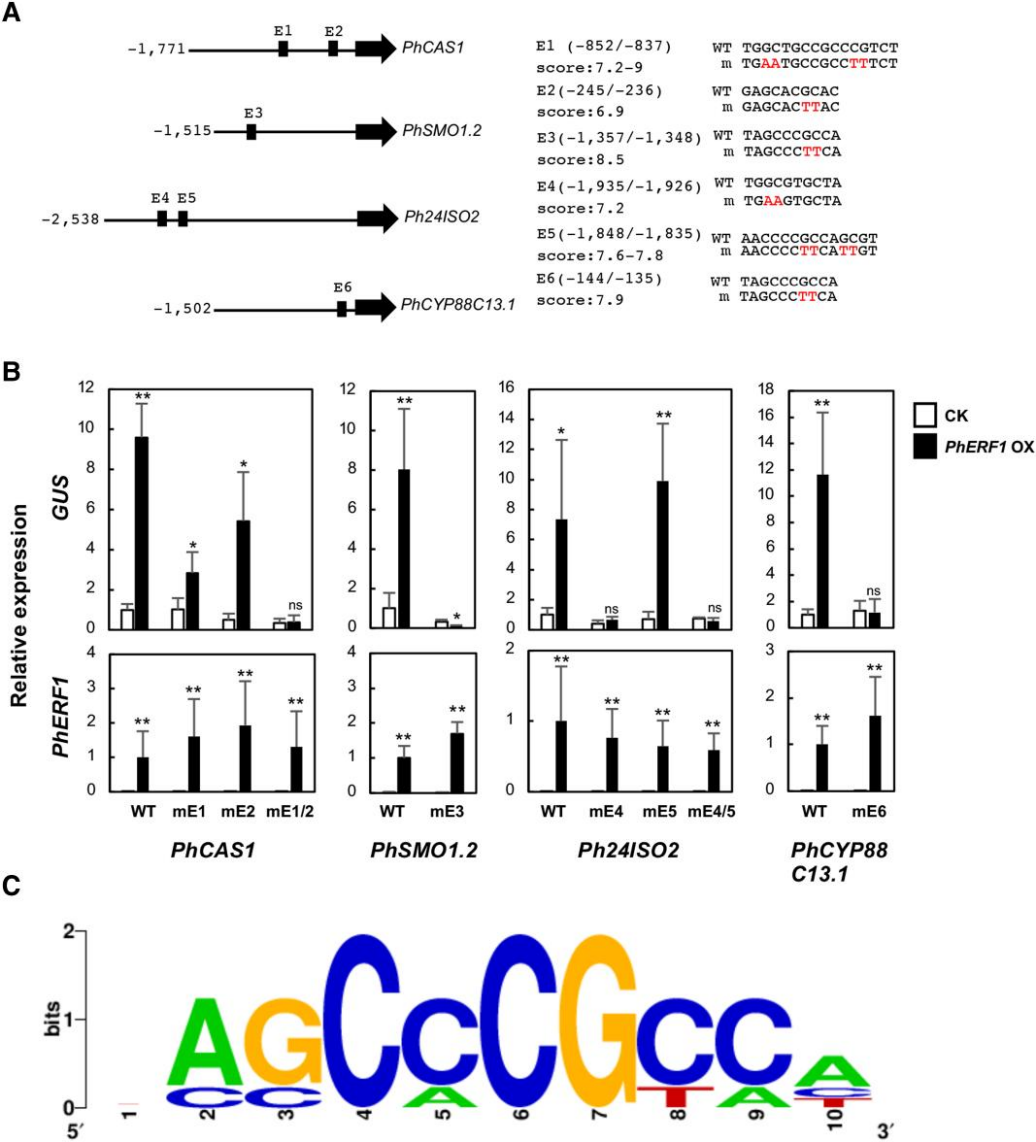
PhERF1 mediates transcriptional regulation of *PhCAS1*, *PhSMO1.2*, *Ph24ISO2*, and *PhCYP88C13.1* through their promoter regions. A) Diagram of the *PhCAS1* (Peaxi162Sch00263g00924), *PhSMO1.2* (Peinf101Sch00284g004025), *Ph24ISO2* (Peaxi162Sch00695g00218), and *PhCYP88C13.1* (Peaxi162Sch00312g00220) promoter regions. Nucleotides were counted from the first ATG. Predicted *cis*-regulatory elements E1 to E6 are indicated as boxes. Note that E1 and E5 are present in multiple partially overlapping copies, each of which is 10 bp in length. Nucleotide sequences of the wild-type (WT) and mutated elements are shown with their positions and scores to the right; mutated nucleotides are in red. B) Transactivation analysis of WT promoter reporters, *PhCAS1pro:GUS*, *PhSMO1.2pro:GUS*, *Ph24ISO2pro:GUS*, and *PhCYP88C13pro:GUS*, and their mutant versions (element [E] names denoted with m). The reporter plasmids were introduced into *N. benthamiana* leaves through *Agrobacterium*-mediated infiltration with either control (CK) pBI101 or *35S:PhERF1* overexpression vector. Transcript levels of the reporter gene *GUS* and *PhERF1* were determined by RT-qPCR. Values are means ± SD of the biological replications. Relative expression levels in the controls were set to 1. For each reporter vector, significant differences relative to the controls were determined by Student's *t*-test; **P < 0.01, *P < 0.05. ns, not significant. C) Sequence logo of the consensus sequence for the *cis*-regulatory elements, E1, E2, E3, E4, and E6, which have been validated experimentally in B. One hundred hypothetical sequences representing the elements were used to create a logo by WebLogo (39). Sequences with the highest scores were used as representatives for E1 and E5.

We asked whether the increased transcript levels of downstream metabolic genes measured by RT-qPCR were caused by direct transcription control by PhERF1 binding to promoter regions. Using the known binding site of NtERF189 (a GC-rich 10-nt element (38)) as a starting point, we computationally predicted putative *cis*-regulatory elements (E1 to E6) recognized by PhERF1 in the promoter sequences of *PhCAS1*, *PhSMO1.2*, *Ph24ISO2*, and *PhCYP88C13.1* (Fig. 5A). E1 and E5 were indeed present as multiple overlapping sequences in these promoters. To test the direct transcriptional activation of the above promoters by PhERF1, we conducted a transient expression assay in *Nicotiana benthamiana* leaves via *Agrobacterium*-mediated infiltration using the *β-glucuronidase* (*GUS*) reporter gene placed under the control of the promoter sequences of *PhCAS1* (−1,771 to −1; counted from the first ATG), *PhSMO1.2* (−1,515 to −1), *Ph24ISO2* (−2,538 to −1), and *PhCYP88C13.1* (−1,502 to −1). We coinfiltrated each GUS reporter construct with EV (CK, as control) or with a construct overexpressing PhERF1; 2 days after infiltration, we harvested samples for RT-qPCR analysis of *GUS* and *PhERF1* relative transcript levels. Compared with the EV controls, relative *GUS* transcript levels were elevated 7.4 to 11 times upon overexpression of *PhERF1* (Fig. 5B), suggesting that PhERF1 mediates the transcriptional activation of the promoter fragments.

To determine whether the predicted *cis*-regulatory elements were required for PhERF1-dependent reporter activation, we mutated highly conserved GC dinucleotides in the elements (17) by PCR-based mutagenesis. We then placed *GUS* under the control of these mutated promoter fragments and repeated the transient transactivation assays. PhERF1 failed to induce the accumulation of *GUS* transcripts when the reporter gene was driven by the mutated *PhSMO1.2* or *PhCYP88C13.1* promoter, which each have one predicted *cis* element (Fig. 5A and B), suggesting that they are required for activation. As the *PhCAS1* promoter harbors two elements, E1 and E2 (Fig. 5A), we mutated them individually and together. The mutated versions of the *PhCAS1* promoter with mutated E1 (mE1) or mE2 showed a significant decrease in relative *GUS* transcript levels, although PhERF1 still induced *GUS* transcription from these mutated promoters. The mutation of both elements (mE1/2) abolished PhERF1-mediated transcriptional activation of the GUS reporter from the *PhCAS1* promoter (Fig. 5B), suggesting that both elements are necessary for promoter activation. The *Ph24ISO* promoter contains two elements, E4 and E5 (Fig. 5A). We detected a complete loss of PhERF1-mediated activation of GUS transcription following mutation of E4, but not E5 (Fig. 5B), indicating that PhERF1 mediates *Ph24ISO* promoter activation by recognizing the promoter element E4. A consensus of the *cis*-regulatory elements (E1, E2, E3, E5, and E6), which have been validated experimentally, is shown as a sequence logo (Fig. 5C).

### Effects of PhERF1 overexpression on steroid metabolism

Given that PhERF1 up-regulates metabolic genes contributing to specialized steroid biosynthesis (Fig. 2A; Table S2), we examined the influence of *PhERF1* overexpression on the accumulation of sterol and related steroidal compounds in petunia leaves. To allow the accumulation of metabolites, we harvested petunia leaves 8 days after *Agrobacterium*-mediated infiltration of the *35S*:*PhERF1* construct. We extracted metabolites and subjected them to gas chromatography–mass spectrometry (GC-MS) and liquid chromatography–mass spectrometry (LC-MS) analyses to measure sterols and specialized steroids, respectively.

**Fig. 6.**
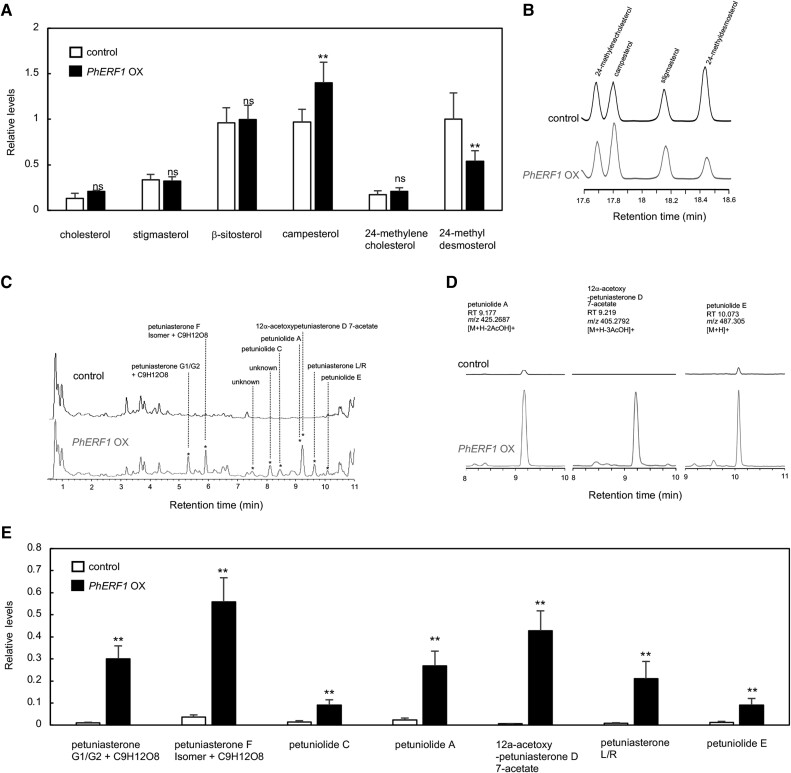
Effects of *PhERF1* overexpression on steroid accumulation in petunia leaves. *PhERF1* was transiently overexpressed via *Agrobacterium-*mediated infiltration in petunia leaves under the control of the 35S promoter (OX). The pBI121 vector was used as the EV control. Leaves were harvested for metabolite analysis 8 days after infiltration. Relative contents of each metabolite were calculated based on area values of the corresponding peaks relative to those of internal standards. Values are means ± SD of biological replicates (*n* = 8 for control, *n* = 18 for *PhERF1* OX). Significant differences relative to the controls were determined by Student's *t*-test. **P < 0.01; ns, not significant. A) Relative levels of cholesterol, campesterol, stigmasterol, β-sitosterol, 24-methylenecholesterol, and 24-methyldesmosterol, as determined by GC-MS analysis. B) TICs of GC-MS analysis. C) TICs of LC-MS analysis. Peaks that were used to calculate the levels shown in E are marked by asterisks. D) EICs of three steroidal compounds. E) Relative levels of petuniolide and petuniasterone derivatives. The area of peaks with highest average intensities for each metabolite was used to calculate their levels.

Specifically, we measured the relative levels of cholesterol, phytosterols (stigmasterol, β-sitosterol, and campesterol), 24-methylenecholesterol, and 24-methyldesmosterol in leaf samples using targeted GC-MS analysis. 24-Methyldesmosterol levels were significantly lower, by 46%, whereas campesterol accumulation increased by 40% with *PhERF1* overexpression compared with EV controls (Fig. 6A and B, Table S7). These changes in sterol levels were in line with those seen for specialized steroids (see below), suggesting an increased metabolic flow to a branch leading to campesterol and specialized steroids and consumption of 24-methyldesmosterol, a product of 24ISO catalysis. This consumption was consistent with the increased expression of downstream genes specific to specialized steroid branches (see Fig. 2A).

We performed LC-MS analysis to detect the specialized steroids petuniolides and petuniasterones. Total ion chromatograms (TICs) revealed many higher peaks and new peaks in extracts obtained from petunia leaves overexpressing *PhERF1* relative to the controls (Fig. 6C, Data Set S5), suggesting a PhERF1-mediated metabolic elicitation. We selected peaks based on similarity (with a tolerance of 0.01) to reported parental ion *m*/*z* values (values reported in (28) and values for metabolites, which were extracted with the keyword Petunia in Biological Source, in the Dictionary of Natural Products [http://dnp.chemnetbase.com]) as well as average intensities of >0.2 in leaves overexpressing *PhERF1* and FC of >10-fold among the samples. We then manually identified these peaks using MS/MS spectra (28) (Fig. S5), since authentic steroid standards were not available. Accordingly, we identified 5 peaks as 3 petuniolide derivatives and 11 peaks as 4 petuniasterone derivatives (Data Set S6). Two of the selected peaks were not assigned to any known metabolites (Data Set S6). Because individual peaks for petuniolide A, 12α-acetoxypetuniasterone D 7-acetate, and petuniolide E were difficult to ascertain in Fig. 6C, we show their extracted ion chromatograms (EICs) (Fig. 6D). We used the area of peaks (marked with asterisks in Fig. 6C) with the highest average values among those for each metabolite to calculate relative accumulation levels (Fig. 6E, Data Set S6). We observed significant increases (6.6- to 67-fold) upon *PhERF1* overexpression for all seven steroidal metabolites (Fig. 6E), indicating that *PhERF1* overexpression induces the accumulation of a group of specialized products.

### PhERF1 overexpression broadly affects metabolism

**Fig. 7.**
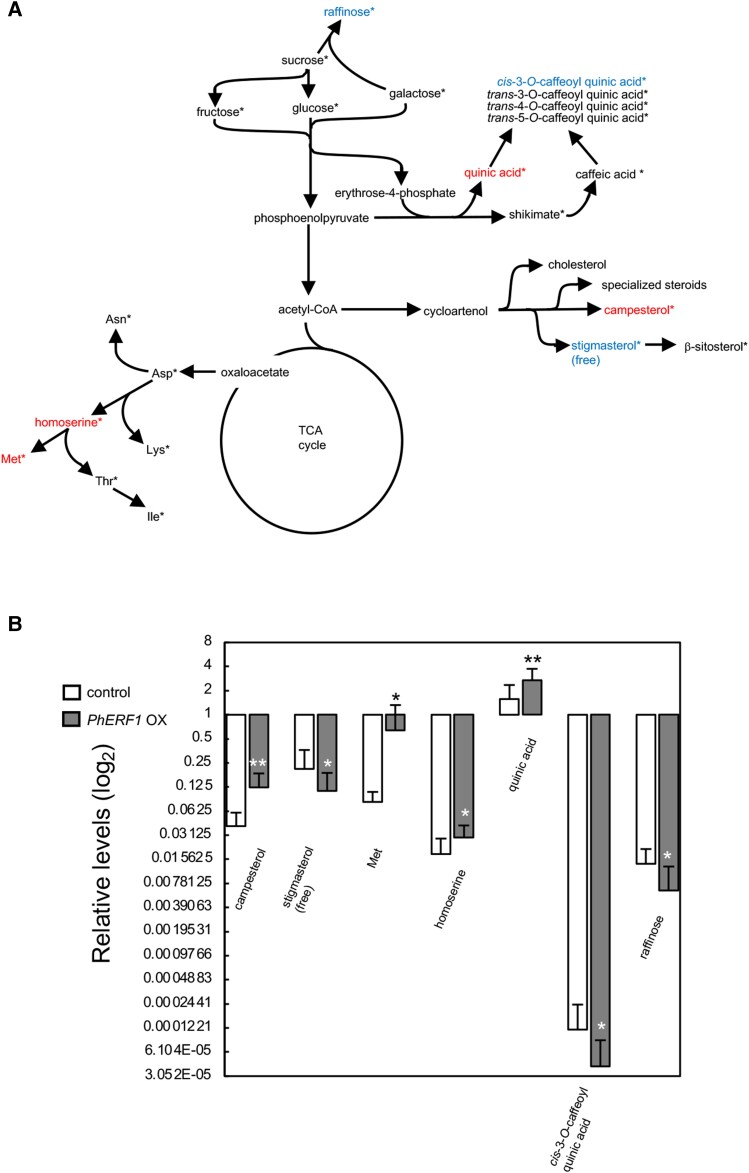
Effects of *PhERF1* overexpression on a wide range of metabolites in petunia leaves. *PhERF1* was transiently overexpressed via *Agrobacterium*-mediated infiltration in petunia leaves under the control of the 35S promoter (OX). The pBI121 vector was used as EV control. Leaves were harvested for comprehensive metabolite analysis 8 days after infiltration. Relative contents for each metabolite were calculated based on area values of the corresponding peaks relative to those of internal standards. A) Metabolic map. All metabolites detected in this analysis are marked by asterisks. Metabolites whose abundance significantly increased or decreased in *PhERF1* OX samples (>1.5-fold) relative to the controls are shown in red and blue, respectively. Significant differences were determined by Student's *t*-test at *P* = 0.05. B) Contents of the increased or decreased metabolites. FC are indicated above the black bars. Values are means ± SD values of biological replicates (*n* = 8 for control, *n* = 18 for *PhERF1* OX). Significant differences relative to the controls were determined by Student's *t*-test. **P < 0.01, *P < 0.05.

To determine whether the PhERF1-mediated transcriptional changes and associated induction of specialized steroids affected primary and secondary metabolism in petunia leaves, we performed comprehensive metabolite profiling by GC-MS. We detected 203 peaks, including 96 identified or annotated metabolites in control leaves and leaves overexpressing *PhERF1*. We detected significant changes of over 50% between the samples only for campesterol, stigmasterol, methionine, homoserine, quinic acid, *cis*-3-*O*-caffeoyl quinic acid, and raffinose among the 96 known metabolites (Fig. 7, Data Set S7). Because of differences between extraction procedures, only free stigmasterol was quantified in Fig. 7, and both free and conjugated forms of the sterol were collectively measured in Fig. 6. We observed a significant decrease for free stigmasterol (Fig. 7) but not for the total forms of the sterol (Fig. 6), when *PhERF1* was overexpressed, suggesting an increase of stigmasterol conjugates due to this overexpression.

### Response of PhERF and metabolic genes to jasmonates

Jasmonate-induced gene expression is a characteristic feature of most homologs of *PhERF* genes in other species (18, 21, 24, 40) but not of most *PhERF* genes (as shown in Fig. 1B). To investigate the response of PhERF1-regulated metabolic genes to jasmonates in petunia leaves, we treated petunia plants with MeJA and examined the expression levels of these genes 24 h later by RNA-seq analysis. We selected DEGs that were up-regulated (over 2-fold) or down-regulated (below 0.5-fold) by the phytohormone treatment (Data Sets S3 and S4). We established that although a significant fraction of the PhERF1-regulated DEGs are induced by MeJA (Fisher's exact test: *P* < 0.001), many PhERF1-regulated DEGs were unaffected (Table S2, Fig. S6).

To better understand the underlying response, we performed a time-course analysis of the response to MeJA for selected *PhERF* and downstream metabolic genes. Accordingly, we measured relative transcript levels using RT-qPCR analysis following MeJA treatment for 1 to 24 h. We observed that MeJA did not induce *PhERF1* expression but significantly increased the expression of *PhERF7* and *PhERF10*, with maximum FC of 8.9- and 4.3-fold, respectively (Fig. S7). We also observed variable and noncoordinated increases in the expression of *PhSMO1.2*, *PhCYP88C13.1*, and *Ph24ISO2* in response to MeJA, whereas the expression of *PhCAS1* was not affected by the phytohormone (Fig. S7). These results suggest that many, but not all, PhERF1-regulated metabolic genes are positively regulated but not necessarily in a coordinated manner in terms of timing or magnitude.

## Discussion

The overexpression of *PhERF1* in petunia leaves led to the transcriptional reprogramming of many sterol biosynthesis and uncharacterized metabolic genes (Fig. 2, Table S2), resulting in the production of a group of specialized steroids, the petuniolides and petuniasterones (Fig. 6). Petuniasterones are characterized by the presence of a ketone group in the A-ring, whereas petuniolides have a spirolactone A-ring structure (41). These ergostane-type steroids have protective properties and may play a crucial role in plant defense (42). Petuniolides have been proposed to act on γ-aminobutyric acid (GABA) receptors in the central nervous system (43) and are generally more toxic to a wide range of insect larvae than petuniasterones (42). The overexpression of *PhERF1* significantly increased the accumulation of three petuniolide derivatives and four petuniasterone derivatives (Fig. 6C–E). Although they did not all reach the thresholds for our selection criteria, we observed an increase in many peaks by LC-MS analysis upon *PhERF1* overexpression (Fig. 6C, Data Set S5). These peaks may correspond to derivatives of known or unknown steroidal compounds. Further analysis will be needed to determine the exact identities and structures of these unknown peaks (Fig. 6C, Data Set S6) to fully understand their potential role.

*PhERF1* overexpression induced the expression of a series of sterol biosynthesis genes, including *24ISO* genes, which contribute to the production of these specialized steroids (Fig. 2, Table S2). The pattern of activated genes in petunia is clearly different from that in tomato, where SlJRE4 activates metabolic genes involved in the cholesterol pathway contributing to steroidal glycoalkaloid production (23, 24, 44), although the sterol and cholesterol pathways are related (Fig. 2A) (31). In this regard, the activity of SMO4 from petunia needs to be experimentally verified, as its tomato homolog suggests that its activity is specific to the cholesterol pathway (31), which may not be consistent with our scenario (Fig. 2A). Furthermore, PhERF1 up-regulated many previously underannotated metabolic genes from the *CYP*, *DOX*, and *AT* families, most forming clusters composed of multiple nonhomologous metabolic genes in the petunia genome (Fig. 3, Table S2). One homologous pair of these clusters (cluster A in Fig. 3) that included *24ISO*, *CYP87*, and *CYP88* genes was reported in a previous study (45). Currently, limited information is available regarding the metabolic pathway downstream of the 24ISO-catalyzed step (45). These downstream steps are responsible for elaborating the steroidal backbone through extensive oxidation and other modifications. It is possible that PhERF1-regulated metabolic genes from the *CYP*, *DOX*, and *AT* families participate in these modification steps, although further research is necessary to elucidate the specific underlying metabolic details. A comparative approach assessing related steroids from a range of plants in the Solanaceae family, such as withanolides from *Withania* species (32) and physalins from *Physalis* species (33), would be beneficial for understanding how different metabolic enzymes contribute to the production of a diverse group of related steroidal compounds. It would be intriguing to investigate whether metabolic gene clusters analogous to those found in petunia exist in the species that produce related steroids. If so, clustering would allow us to pinpoint the relevant genes involved in the pathways.

We hypothesize that the metabolic effects of *PhERF1* overexpression are primarily related to the production of specialized steroids. This assumption is supported by the enrichment of DEGs induced by *PhERF1* in pathways related to the biosynthesis of steroidal compounds, rather than in other metabolic pathways (Tables S2 and S4). Comprehensive metabolite profiling further supports this idea, as few metabolites not related to the sterol biosynthesis pathway were significantly affected in their abundance by *PhERF1* overexpression (Fig. 7). A substantial portion of the alterations observed in the levels of nonsterol metabolites could potentially result from indirect effects stemming from the redirection of metabolic flux caused by the ongoing metabolic changes, rather than being directly attributed to the PhERF1 transcription factor. The significant 7.8-fold increase of methionine (Fig. 7B) cannot be attributed to changes in the expression of genes directly involved in amino acid accumulation (Table S2). Similarly, overexpression and mutational loss of *NtERF189* in tobacco have significant effects on the accumulation of metabolites not involved in the nicotine biosynthetic pathway (13). The metabolomic changes detected in tobacco do not necessarily overlap with those induced by *PhERF1* overexpression in petunia. For example, methionine was not significantly affected by transient overexpression of *NtERF189* in *N. benthamiana* leaves (13). The distinct metabolic changes induced by related ERFs in petunia and tobacco (13) suggest different mechanisms of metabolic regulation. A large, sustained change in the level of even a single amino acid may adversely affect plant growth and development and therefore may only be tolerable in a transient expression system. This notion underscores the potential of transient systems over the generation of conventional stable transgenic lines for economically viable plant-based bioproduction of specialized products.

Small groups of evolutionarily conserved *ERF* genes often form clusters in plant genomes (18). Unlike clusters of nonhomologous metabolic genes (46), *ERF* clusters consist of multiple homologous genes arranged in tandem, potentially resulting from repeated gene duplication. In *ERF* clusters from Solanaceae species, *ERF* genes at the outer edge of the clusters are relatively well conserved, whereas those in the central region are more variable among species (Fig. 1A and C). This pattern suggests that these clusters expanded in distinct lineages primarily through the addition of genes at the centers of clusters. *PhERF1*, *PhERF2*, and their homoeologs, specific to the petunia lineage, are considered the petunia counterparts of *NtERF189* and *SlJRE4*, based on their genomic place within the central variable region of the clusters and their high expression (Fig. 1B and C). This notion is supported by the fact that PhERF1 and PhERF2 have higher transactivation activity than related regulators. The comparable expression levels (Fig. 1B) and transactivation activities (Fig. 4) of PhERF1 and PhERF2 suggest their functional similarity as regulators of specialized steroids, with potential redundancy as with the multiple CrORCAs regulating alkaloid biosynthesis in *C. roseus* (20).

In contrast to the reported homologs from other species, the expression of most *PhERF* genes, including *PhERF1* and *PhERF2*, is not induced by jasmonates (Fig. 1B, Fig. S7). Although MeJA regulated a fraction of PhERF1-regulated metabolic genes (Fig. S6), we did not detect coordinated induction of gene expression (Fig. S7), supporting the idea that PhERF1 may not play a significant role in phytohormonal regulation. Further investigation will be required to determine whether and how the defense-related phytohormone regulates the biosynthesis of bioactive steroids in *Petunia* species, possibly through a PhERF1-independent mechanism. The differences in response to jasmonates among ERF regulators from different species suggest a regulatory change during speciation.

Our study shows that overexpressing the transcriptional regulator gene *PhERF1* in petunia activated sterol biosynthesis and the expression of several mostly clustered metabolic genes, leading to the production of defense-related steroids. This approach of using evolutionarily conserved factors as genetic tools to stimulate the production of bioactive natural products, which has previously been proposed and demonstrated (18, 47, 48), has significant potential for bioproduction purposes, even in the absence of prior knowledge of the metabolic pathways involved. We plan to further examine the regulatory functions of the other *ERF* genes in the clade to fully test the notion that evolutionary proximity indeed results in the control of similar metabolic pathways.

## Materials and methods

### Plant growth and treatment

Surface-sterilized seeds of *P. hybrida* cv. Mitchell diploid (W115) and *N. benthamiana* were germinated on half-strength Gamborg B5 medium solidified with 0.7% (*w*/*v*) agar and supplemented with 3% (*w*/*v*) sucrose. Two-week-old seedlings were transferred to soil (JA Nipi Horticulture Soil No. 1; Nippon Hiryo, Gunma, Japan) in pots and grown in a culture room at 25°C in a 16-h light/8-h dark photoperiod. Four-week-old petunia and 6-week-old *N. benthamiana* plants were used for the experiments. The topmost fully expanded leaves at comparable positions on the plants were employed in each experiment.

The plants were exposed to MeJA gas in 12-L air-tight plastic buckets. A piece of paper towel soaked with 1.2 mL of a volatile liquid form of MeJA (Fujifilm Wako, Osaka, Japan) was placed in the bucket with two individual plants.

### Plasmid construction

Genomic DNA was isolated from petunia leaves with the cetyltrimethylammonium bromide (CTAB) method (49). The full-length coding region of the intron-less *PhERF1* gene (Peaxi162Scf00672g00031) and the promoter regions of *PhCAS1* (Peaxi162Sch00263g00924), *PhSMO1.2* (Peinf101Sch00284g004025), *Ph24ISO2* (Peaxi162Sch00695g00218), and *PhCYP88C13.1* (Peaxi162Sch00312g00220) were amplified by PCR using genomic DNA as a template and high-fidelity PrimeSTAR Max DNA polymerase (Takara, Kyoto, Japan). The full-length coding sequences of *PhERF2* (Peaxi162Scf01094g00002), *PhERF5* (Peaxi162Scf00475g00077), and *PhERF7* (Peaxi162Scf00475g00053) were synthesized (Eurofins Genomics, Tokyo) and amplified by PCR. The amplified fragments were cloned into pENTR/D-TOPO (Thermo Fisher Scientific, Waltham, MA). Mutations were introduced in the *cis*-regulatory elements by PCR-based mutagenesis (50). After the sequences were confirmed by Sanger sequencing, the coding and promoter regions in pENTR/D-TOPO were recombined into pGWB2, pGWB417, and pGWB5 vectors (37, 51) using LR Clonase II (Thermo Fisher Scientific) to generate binary vectors.

### Computational analysis

A BLASTP search was conducted to identify petunia proteins involved in sterol biosynthesis (Table S6) using tomato counterparts (Table S5) as queries and petunia ATs, CYPs, and DOXs included in phylogenetic trees (Figs. S2–S4) using corresponding family proteins encoding DEGs in clusters (Fig. 3) as queries. All proteins predicted in the *P. axillaris* (v.1.6.2) and *P. inflata* (v.1.0.1) genomes were included in the search. Cutoff e-values were set to <1e^−180^, < 1e^−100^, and <1e^−50^ for query proteins of ≥300, 299 to 201, and ≤200 amino acids, respectively.

The full-length amino acid sequences of the identified proteins were aligned using MUSCLE (52), and an unrooted phylogenetic tree was reconstructed using the neighbor-jointing algorithm with MEGAX (53) using default settings.

To search for significantly enriched GO terms among up-regulated or down-regulated DEGs upon *PhERF1* overexpression relative to all genes predicted in either *P. axillaris* or *P. inflata* genomes, the Plant Transcriptional Regulatory Map (http://plantregmap.gao-lab.org/go.php) (54) was used with a *P*-value threshold set to 0.001.

Putative *cis*-regulatory elements recognized by PhERF1 were predicted in promoter sequences with a position weight matrix representing NtERF189 binding specificity (38) using Regulatory Sequence Analysis Tools (http://rsat.sb-roscoff.fr/matrix-scan-quick_form.cgi) (55) with default settings. The elements E1 to E6 (Fig. 6A) were detected with scores of 6.9 to 9.

### Agrobacterium-mediated transient overexpression

Several vectors were used, including pGWB2 and pGWB417, for the individual overexpression of *PhERF1*, *PhERF2*, *PhERF5*, *PhERF7*, *SlJRE4* (Solyc01g090340), and *NtERF189* (GenBank: AB827951). pGWB5-based promoter reporter vectors were also generated for the *PhCAS1*, *PhSMO1.2*, *Ph24ISO2*, and *PhCYP88C13.1* promoter fragments; the control vectors were pBI121 and pBI101. Additionally, the p19 vector was included for all *Agrobacterium* (*Agrobacterium tumefaciens*)–mediated infiltrations for silencing suppression (56). The pGWB417-based *NtERF189* overexpression vector was generated previously (22). The vectors were introduced into *Agrobacterium* strain GV3101 by heat shock treatment. The various coding regions were individually transiently heterologously overexpressed in the youngest fully expanded leaves of plants as described (57). Bacterial suspensions of the *ERF* overexpression vector or the control vector pBI121 and p19 vector were combined at a ratio of 7:3 (*v*/*v*), and those for *ERF* overexpression or the control vector pBI101, promoter reporter vectors, and the p19 vector were mixed at a ratio of 7:7:6 (*v*/*v*/*v*). The leaves were harvested 2 days after infiltration for RNA and protein extraction and 8 days after infiltration for metabolite extraction.

### RNA-seq analysis

Total RNA was isolated using a Plant RNeasy kit with DNase I treatment (Qiagen, Venlo, Netherlands). The cDNA libraries were constructed using MGIEasy RNA Library Prep Set (MGI, Shenzhen, China), purified using AMPure XP beads (Beckman Coulter, Beverly, MA), validated on an Agilent 2100 Bioanalyzer (Agilent, Santa Clara, CA), and sequenced in a paired-end manner using a DNBSEQ-G400 instrument (BGI, Wuhan, China). Low-quality reads (those with >20% of bases with a quality score <10) and reads with unknown bases (more than 5% N bases) were removed, and sequences derived from adaptors were trimmed. The clean reads were mapped to the reference genomes of *P. axillaris* v.1.6.2 and *P. inflata* v1.0.1 using Bowtie2 (v.2.2.5; http://bowtie-bio.sourceforge.net/Bowtie2/index.shtml) with the following parameters: -q --phred33 --sensitive --dpad 0 --gbar 99999999 --mp 1,1 --np 1 --score-min L,0, -0.1 -I 1 -X 1000 --no- mixed --no-discordant -p 1 -k 200. Gene expression levels were calculated with RSEM (v.1.2.12; http://deweylab.biostat.wisc.edu/ RSEM) with default parameters. DEGs were identified using DEseq2 (58) with a FC of normalized expression values >2 or <0.5 and adjusted *P*-values below 0.05. Three and two biological replicates were used for each sample in *PhERF1* overexpression and MeJA experiments, respectively.

### RT-qPCR analysis

RNA isolation, RT, and qPCR were performed as described (21). *EF1α* (Peaxi162Scf00351g00312) was used as a reference to normalize expression levels. Primer sequences are in Table S8. Since primer specificities were not validated experimentally, the possibility of amplification of closely related sequences cannot be excluded.

### Immunoblot analysis

Protein extraction, separation by electrophoresis, transfer to membranes, membrane incubation with antibodies and washes, and chemiluminescence detection were carried out as described (22). An anti-Myc tag polyclonal antibody (PA1-981; Thermo Fisher Scientific) and goat horseradish peroxidase–conjugated anti-rabbit IgG (H + L) (Thermo Fisher Scientific) were diluted at 1:2,000 and 1:10,000 and used as primary and secondary antibodies, respectively. The intensities of the bands corresponding to the Myc-tagged fusion proteins were quantified using ImageQuant TL software (GE Healthcare, Chicago, IL). These intensities were then utilized to normalize the relative expression levels of the *PhERF1*-regulated metabolic genes, which were determined through RT-qPCR analysis.

### Metabolite analysis

Leaf tissues were ground into a fine powder with a mortar and pestle, and metabolites were extracted for LC-MS analysis as described (59). The extracts were analyzed using LC-QTOF-MS (LC, Waters Acquity UPLC system; MS, Waters Xevo G2 Q-Tof; Waters, Milford, MA). LC separation, MS detection, and MS/MS data acquisition were conducted as described (59) with the polarity of electrospray ionization applied in positive ionization mode. Data processing was conducted using MS-DIAL ver. 4.70 (http://prime.psc.riken.jp/compms/msdial/main.html) (60).

For GC-MS analysis targeting sterols, extraction was conducted as previously described (45). Dry residues from tissues (25 mg fresh weight) were dissolved in 50 µL *N*-methyl-*N*-trimethylsilyl trifluoroacetamide containing 0.09 µg 5-α-cholestane (Nacalai, Kyoto, Japan). Metabolites were separated and quantified with a gas–liquid chromatograph (GCMS-TQ8040; Shimadzu, Kyoto, Japan) equipped with a DB-1 (30 m × 0.25 mm, 0.25 μm film thickness; Agilent) capillary column using a thermal gradient of 80°C for 1 min followed by a temperature increase to 300°C at a rate of 20°C/min and held at 300°C for 10 min, as previously described (61). Cholesterol (Fujifilm Wako), campesterol, stigmasterol, and β-sitosterol (Tama Biochemical, Tokyo); 24-methylenecholesterol (Avanti Polar Lipids, Birmingham, AL); and 24-methyldesmosterol, which was produced through a reaction catalyzed by recombinant Ws24ISO from winter cherry (*Withania somnifera*) in yeast strain T21 (45), were used as authentic standards.

Comprehensive GC-MS analysis, metabolite extraction, addition of ten internal standards, derivatization, GC separation, and MS detection were performed as previously described (62). Chromatograms and mass spectra were preprocessed using the high-throughput data analysis (HDA) method (63). Normalization was performed using the peak area of hexadecanoate-^13^C_4_.

## Accession numbers

RNA-seq data are available at SRA under accession numbers DRA015271 to DRA015276 from PRJDB14882 and DRA015277 to DRA015280 from PRJDB14898.

## Supplementary Material

pgad326_Supplementary_Data

## Data Availability

All data are included in the manuscript and/or supporting information.
