# Supplementary material for: Induced production of specialized steroids by transcriptional reprogramming in Petunia hybrida
Source: PNAS Nexus. 2023 Oct 31;2(10):pgad326. doi: 10.1093/pnasnexus/pgad326 (PMC10619512; doi:10.1093/pnasnexus/pgad326)
Supplement: pgad326_Supplementary_Data [file pgad326_supplementary_data.zip › PNASNEXUS-PNASNEXUS-2023-00661RR-s07.pdf]

Supporting Information for

Induced production of specialized steroids by transcriptional reprogramming in *Petunia hybrida*

Tsubasa Shoji, Satoko Sugawara, Tetsuya Mori, Makoto Kobayashi, Miyako Kusano, and Kazuki Saito

Tsubasa Shoji
Email: tsubasa@inm.u-toyama.ac.jp

This PDF file includes:

Supporting text
Figures S1 to S7
Tables S1 to S8

Other supporting materials for this manuscript include the following:

Datasets S1 to S7

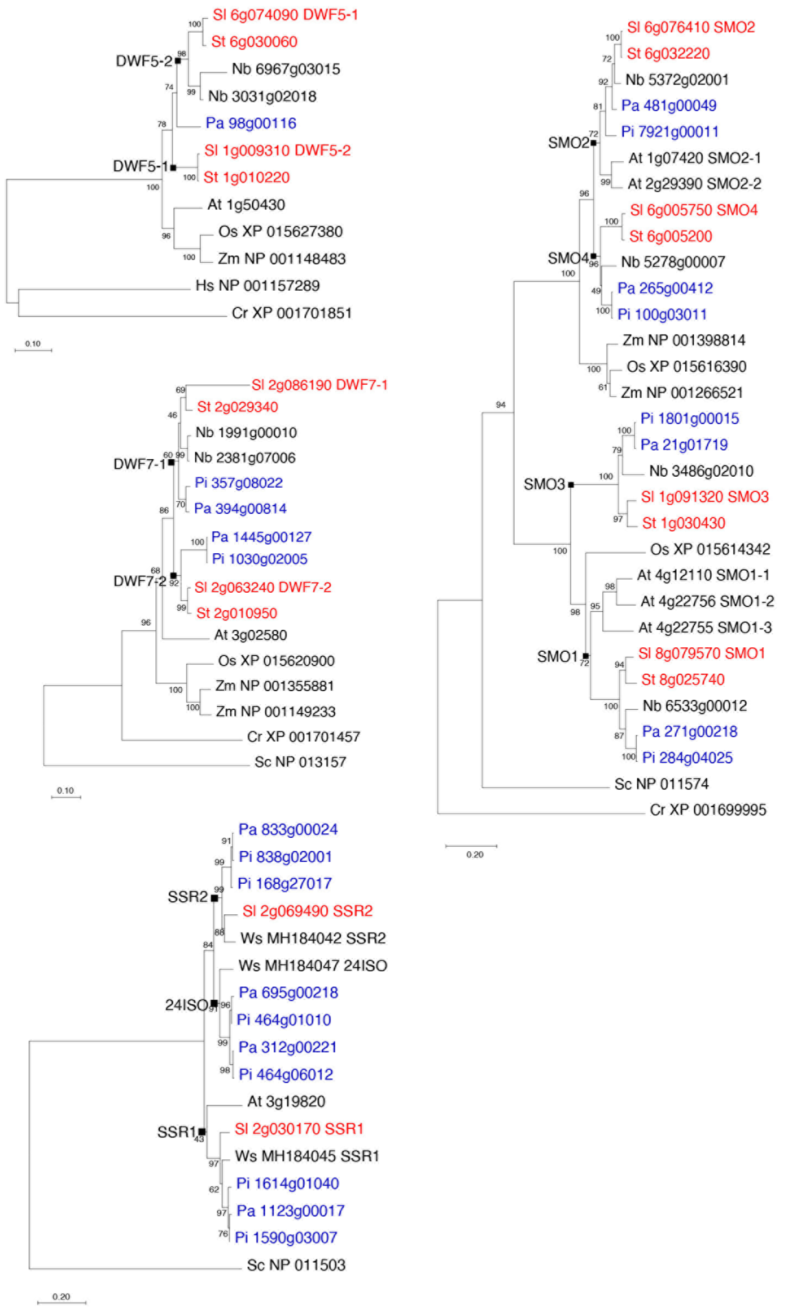

Fig. S1. Phylogenetic analysis of Dwarf 5 (DWF5), Dwarf 7 (DWF7), sterol methyl oxidase (SMO), sterol side reductase (SSR), sterol D24-isomerase (24ISO), and related proteins from petunia and other species. Gene model IDs and accession numbers are indicated. Proteins from *P. axillaris* (Pa; blue), *P. inflata* (Pi; blue), tomato (Sl; *Solanum lycopersicum*; red), potato (St; *Solanum tuberosum*; red), *Nicotiana benthamiana* (Nb), ashwagandha (Ws; *Withania somnifera*), Arabidopsis (At; *Arabidopsis thaliana*), rice (Os; *Oryza sativa*), maize (*Zea mays*) *Saccharomyces cerevisiae* (Sc), *Chlamydomonas reinhardtii* (Cr), and *Homo sapiens* (Hs) are included. Branch points for the indicated subgroups are marked with black squares. The percentage support from 1,050 bootstrap replicates is indicated at branch nodes. The scale bars indicate the number of amino acid substitutions per site.

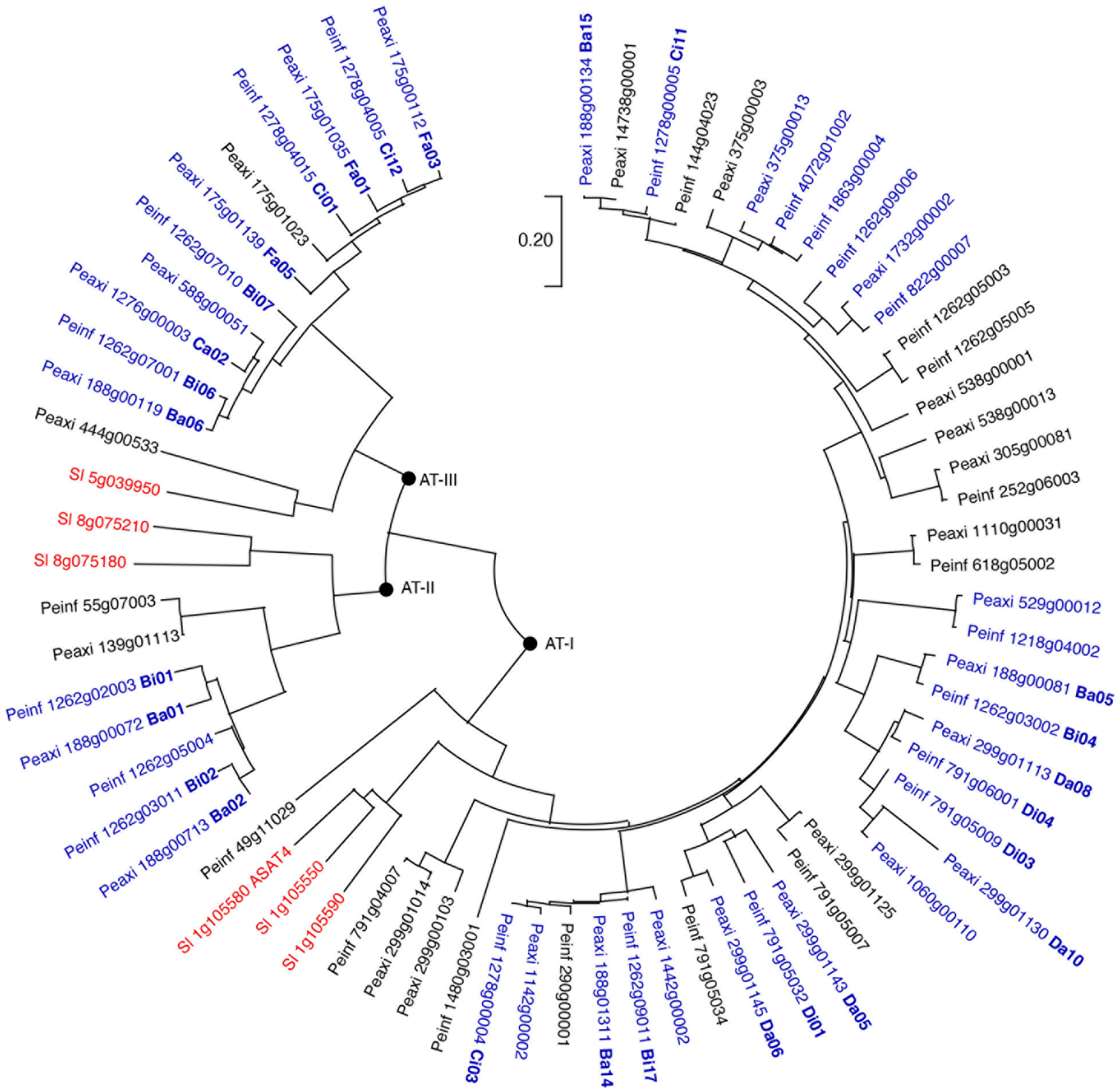

Fig. S2. Phylogenetic analysis of BAHD-type acyltransferase (AT) family proteins from petunia and tomato. Proteins from *P. axillaris* (Peaxi), *P. inflata* (Peinf), and tomato (Sl; red) are included in the tree. Petunia proteins encoded by genes that were upregulated by PhERF1 are shown in blue. Labels indicating the clusters and positions (see **Fig. 3**) are included when applicable. Branch points of clades for subgroups AT-**I**, AT-**II**, and AT-**III** are marked with black circles. The scale bar indicates the number of amino acid substitutions per site.

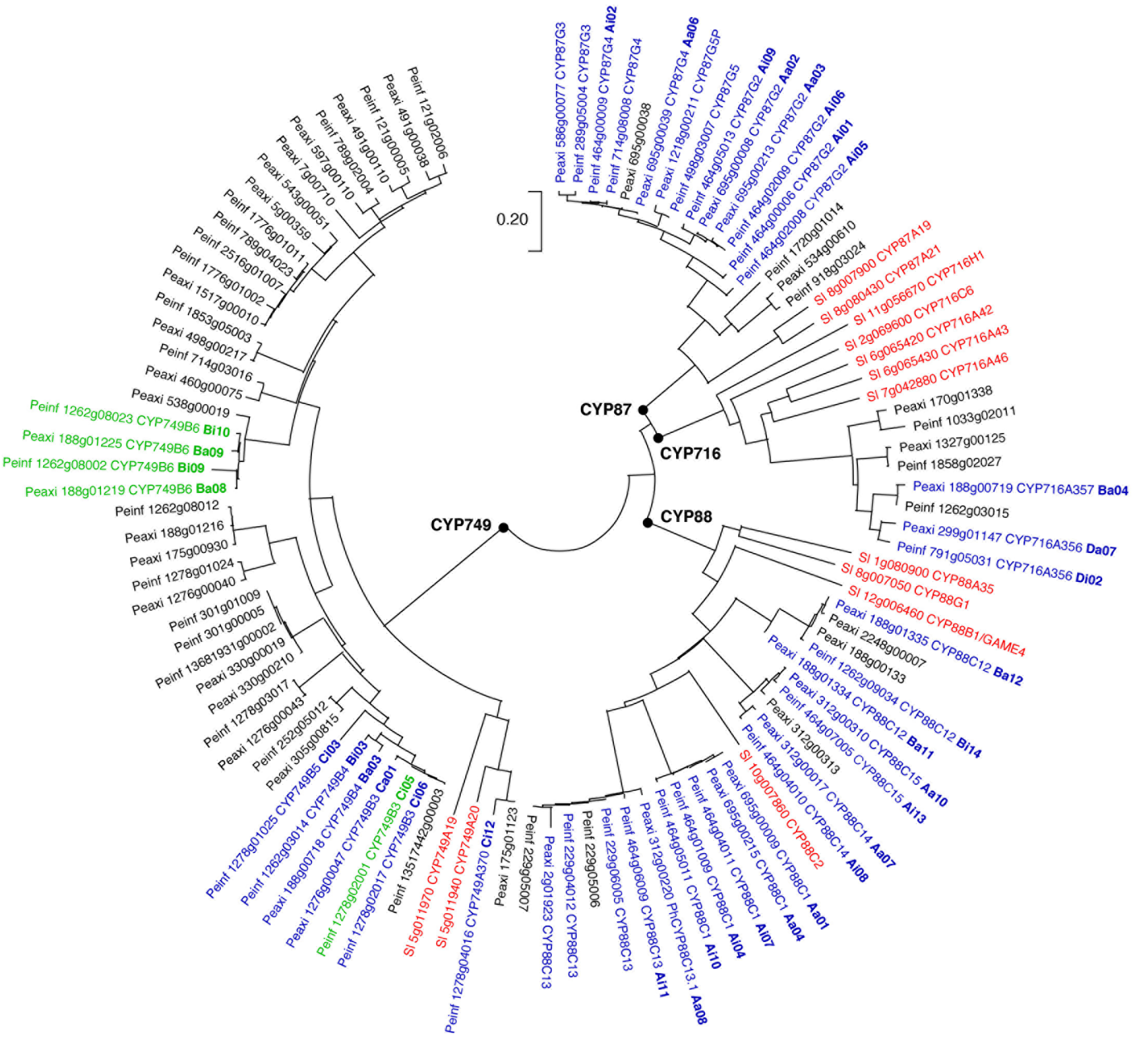

Fig. S3. Phylogenetic analysis of CYP87, CYP88, CYP716, and CYP479 family proteins from petunia and tomato. Proteins from *P. axillaris* (Peaxi), *P. inflata* (Peinf), and tomato (Sl; red) are included in the tree. Petunia proteins encoded by genes that were upregulated or downregulated by PhERF1 are shown in blue and green, respectively. Labels indicating clusters and positions (see **Fig. 3**) are shown when applicable. Branch points of clades for indicated CYP families are marked with black circles. The scale bar indicates the number of amino acid substitutions per site.

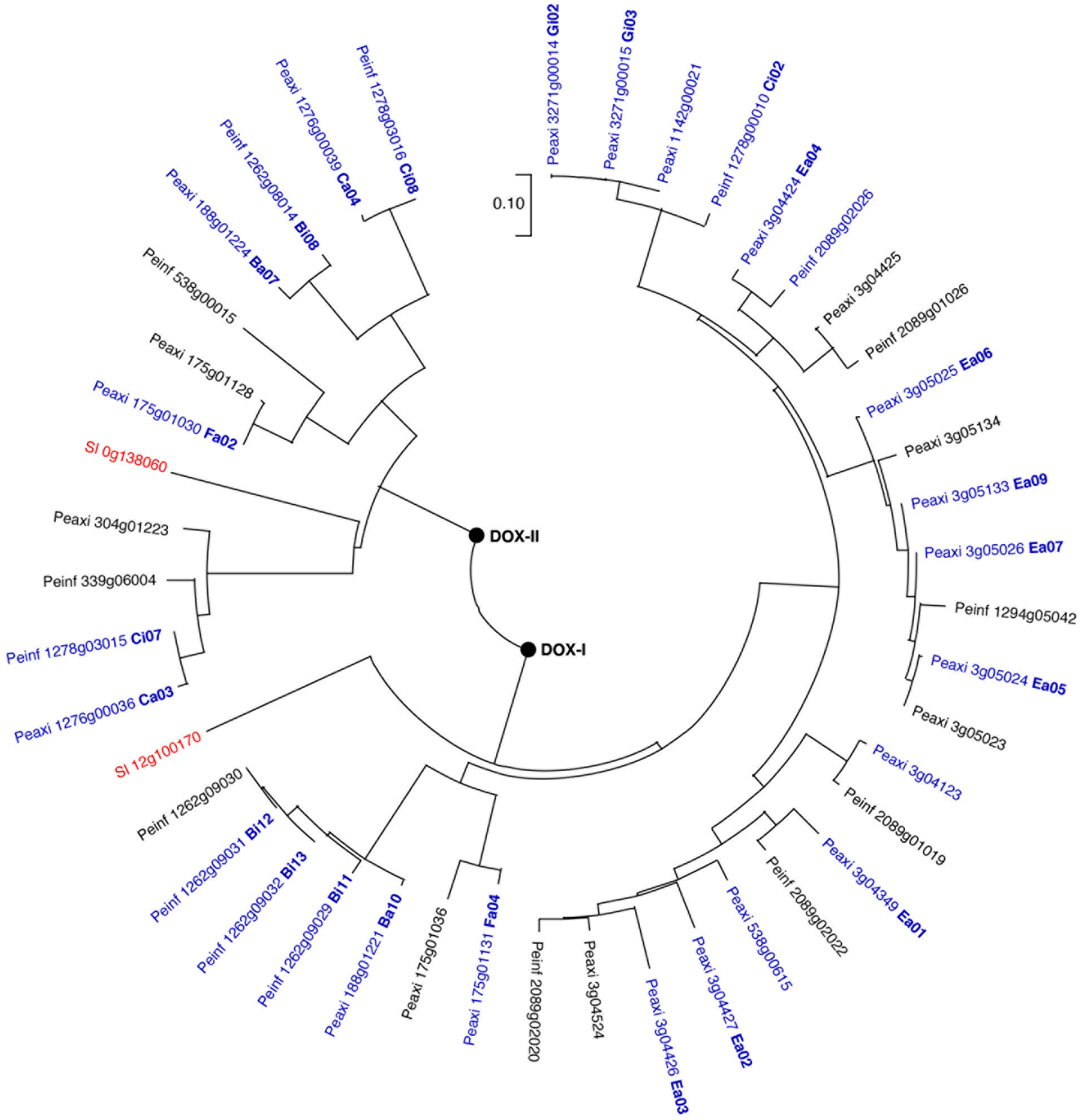

Fig. S4. Phylogenetic analysis of 2-oxoglutarate-dependent dioxygenase (DOX) family proteins from petunia and tomato. Proteins from *P. axillaris* (Peaxi), *P. inflata* (Peinf), and tomato (Sl; red) are included in the tree. Petunia proteins encoding genes that were upregulated by *PhERF1* are shown in blue. Labels indicating clusters and positions (see **Fig. 3**) are shown when applicable. Branch points of clades for subgroups DOX-I and DOX-II are indicated with black circles. The scale bar indicates the number of amino acid substitutions per site.

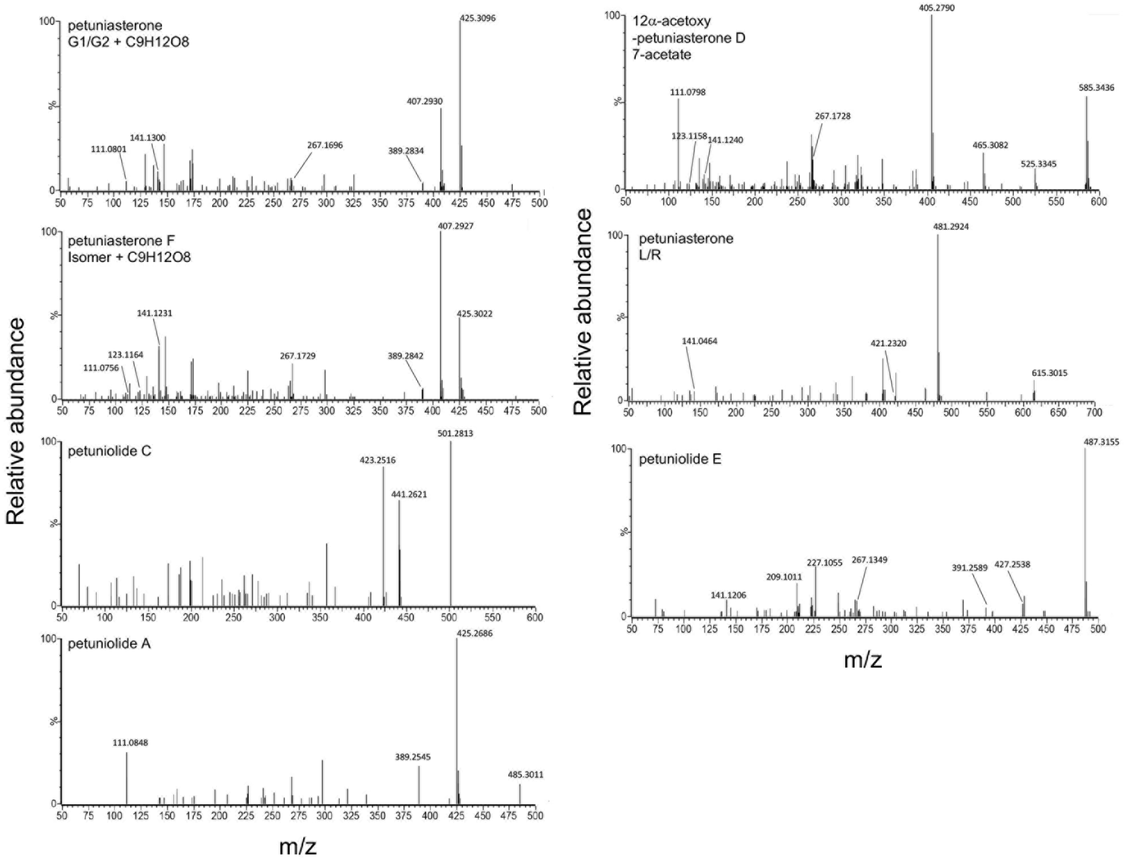

Fig. S5. MS/MS spectra of steroidal metabolites. Precursor and fragment ions and their corresponding *m*/*z* values are indicated as the basis for chemical identification.

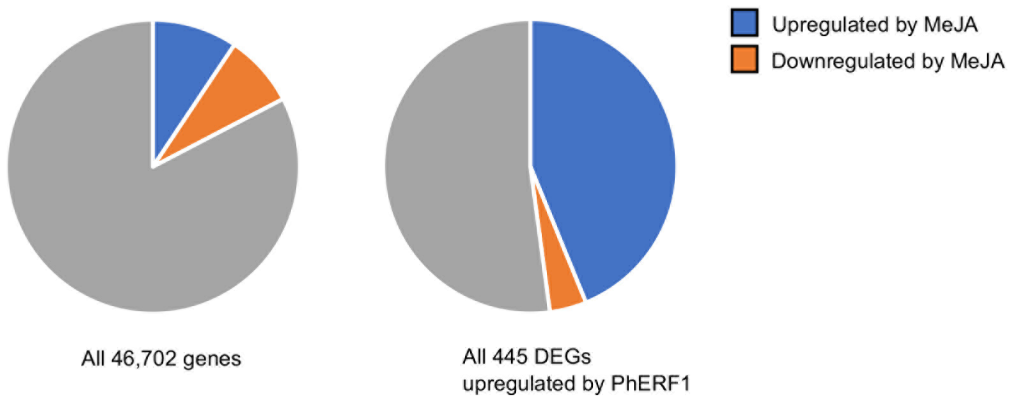

Fig. S6. Proportion of genes regulated by methyl jasmonate (MeJA) in DEGs upregulated by PhERF1. The *P*-values resulting from Fisher's exact test for genes upregulated (blue) or downregulated (orange) by MeJA in the DEGs are both <0.001, indicating significant increase and decrease of the respective gene sets.

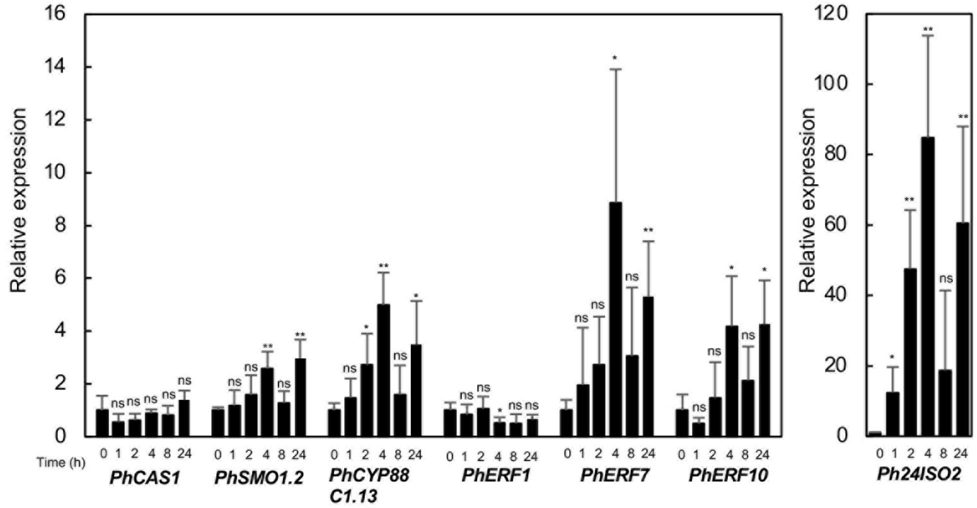

Fig. S7. Response of *PhCAS1*, *PhSMO1.2*, *Ph24ISO2*, *PhCYP88C13.1*, *PhERF1*, *PhERF7*, and *PhERF10* expression to methyl jasmonate (MeJA) in petunia. Transcript levels were determined by RT-qPCR analysis in the leaves. Values are means ± SD for biological replicates. The plants were treated with MeJA for 0, 1, 2, 4, 8, or 24 h. Significant differences relative to 0-h levels were determined by Student's *t*-test. ** $P < 0.01$, * $P < 0.05$; ns, not significant.

Table S1 Expression data of *ERF* genes in petunia

		PhERF1 overexpression (OX) experiment				Methyl jasmonate (MeJA) treatment experiment			
		Normalized expression value				Normalized expression value			
Gene model ID	Gene name	Control	*PhERF1* OX	Log_2FC	Significant Up/Down	Control	MeJA	Log_2FC	Significant Up/Down
Peaxi162Scf00672g00031	*PhERF1*	65.49	14950.34	7.83	Up	8.57	2.64	-1.70	ns
Peinf101Scf00094g12001	*PhERF1L1*	58.25	13160.57	7.82	Up	7.16	3.67	-0.96	ns
Peaxi162Scf00475g00071	*PhERF1L2*	0.66	56.90	6.43	Up	0.00	0.00	0.00	ns
Peinf101Scf00094g14002	*PhERF2L2*	0.35	2.24	2.69	ns	0.35	0.14	-1.32	ns
Peaxi162Scf01094g00002	*PhERF2*	86.13	68.25	-0.34	ns	22.07	12.65	-0.80	ns
Peinf101Scf00094g11001	*PhERF2L1*	76.84	66.75	-0.20	ns	19.73	9.42	-1.07	ns
Peinf101Scf02665g00004	*PhERF3L2*	0.00	0.00	0.00	ns	0.00	0.00	0.00	ns
Peaxi162Scf00475g00064	*PhERF3*	0.34	0.34	0.01	ns	0.15	0.90	2.60	ns
Peinf101Scf00546g00003	*PhERF3L1*	0.23	0.12	-0.94	ns	0.15	0.90	2.59	ns
Peinf101Scf00221g06003	*PhERF4*	0.00	0.00	0.00	ns	0.00	0.00	0.00	ns
Peaxi162Scf00475g00077	*PhERF5*	0.11	0.21	0.97	ns	0.00	0.00	0.00	ns
Peinf101Scf02665g00005	*PhERF5L1*	0.00	0.00	0.00	ns	0.00	0.00	0.00	ns
Peaxi162Scf00475g00061	*PhERF6*	0.00	0.00	0.00	ns	0.13	0.40	1.61	ns
Peinf101Scf00546g01009	*PhERF6L1*	0.00	0.00	0.00	ns	0.13	0.39	1.59	ns
Peaxi162Scf00475g00053	*PhERF7*	0.00	0.00	0.00	ns	0.17	7.23	5.42	ns
Peaxi162Scf00030g00022	*PhERF8*	8.47	0.66	-3.68	ns	0.00	0.00	0.00	ns
Peaxi162Scf00030g00410	*PhERF9*	1.65	5.00	1.60	ns	0.00	0.00	0.00	ns
Peinf101Scf00094g09004	*PhERF9L1*	1.64	3.72	1.18	ns	0.00	0.00	0.00	ns
Peaxi162Scf00199g00056	*PhERF10*	0.00	0.00	0.00	ns	0.17	12.98	6.25	Up
Peinf101Scf01605g08001	*PhERF10L1*	0.00	0.00	0.00	ns	0.17	7.17	5.40	na

Note: Gene models IDs of *Petunia axillaris* are colored in green. FC; fold change, ns; not significant.

Table S2 Expression data of PhERF1-upregulated genes

No.	Gene name or family	Label	Gene model ID	*PhERF1* overexpression (OX) experiment: Normalized expression value: Control	*PhERF1* OX	Log$_2$FC	Response to MeJA	Description
1	*PhERF1*		Peaxi162Scf00672g00031	65.49	14950.34	7.83	ns	ERF transcription factor
2	*PhERF1L1*		Peinf101Scf00094g12001	58.25	13160.57	7.82	ns	ERF transcription factor
3	*PhERF1L2*		Peaxi162Scf00475g00071	0.66	56.90	6.43	ns	ERF transcription factor
4	*PhHMGR3*		Peaxi162Scf00861g00321	2905.37	6599.29	1.18	Up	3-hydroxy-3-methylglutaryl-coenzyme A reductase
5	*PhHMGR6*		Peinf101Scf00409g05014	2778.38	6384.43	1.20	Up	3-hydroxy-3-methylglutaryl-coenzyme A reductase
6	*PhSQO3*		Peaxi162Scf01161g00325	2084.06	4307.47	1.05	ns	squalene monooxygenase
7	*PhSQO6*		Peinf101Scf00736g22027	1951.35	4096.82	1.07	ns	squalene monooxygenase
8	*PhCAS1*		Peaxi162Scf00263g00924	1411.93	4845.27	1.78	Up	cycloartenol synthase
9	*PhCAS6*		Peinf101Scf00172g00004	1303.25	4388.45	1.75	Up	cycloartenol synthase
10	*PhSMT1.2*		Peinf101Scf02980g00010	1880.37	7316.79	1.96	Up	sterol methyltransferase
11	*PhSMO1.1*		Peaxi162Scf00271g00218	543.72	2400.87	2.14	Up	sterol C4-methyl oxidase 1-2
12	*PhSMO1.2*		Peinf101Scf00284g04025	539.53	2382.79	2.14	Up	sterol C4-methyl oxidase 1-2
13	*PhERG28.1*		Peaxi162Scf00628g00517	159.18	330.92	1.06	Up	integral membrane family protein
14	*PhERG28.2*		Peinf101Scf00736g11022	158.51	331.65	1.07	Up	integral membrane family protein
15	*PhCPI1*		Peaxi162Scf00378g00519	324.08	936.14	1.53	Up	cyclopropyl isomerase
16	*PhCPI6*		Peinf101Scf00526g04007	264.82	784.16	1.57	Up	cyclopropyl isomerase
17	*PhCYP51G.1*		Peaxi162Scf00097g00139	3048.65	6404.97	1.07	Up	lanosterol 14-alpha demethylase
18	*PhCYP51G.4*		Peinf101Scf03622g00016	2866.15	5979.96	1.06	Up	lanosterol 14-alpha demethylase
19	*PhSMO4.1*		Peaxi162Scf00265g00412	693.93	6139.29	3.15	Up	sterol 4-alpha-methyl-oxidase 2-2
20	*PhSMO4.2*		Peinf101Scf00100g03011	763.91	7066.43	3.21	Up	sterol 4-alpha-methyl-oxidase 2-2
21	*PhDWF7-1.1*		Peaxi162Scf01445g00127	3613.45	9654.86	1.42	Up	delta(7)-sterol-C5(6)-desaturase
22	*PhDWF7-1.2*		Peinf101Scf01030g02005	3497.35	9327.82	1.42	Up	delta(7)-sterol-C5(6)-desaturase
23	*PhDWF5.1*		Peaxi162Scf00098g00116	3942.26	10688.10	1.44	Up	7-dehydrocholesterol reductase
24	CYP88C1	Aa01	Peaxi162Scf00695g00009	207.35	6672.48	5.01	Up	cytochrome P450 superfamily protein
25	CYP87G2	Aa02	Peaxi162Scf00695g00008	60.11	6196.11	6.69	Up	cytochrome P450 superfamily protein
26	CYP87G2	Aa03	Peaxi162Scf00695g00213	52.25	4971.66	6.57	Up	cytochrome P450 superfamily protein
27	CYP88C1	Aa04	Peaxi162Scf00695g00215	404.83	5311.63	3.71	Up	cytochrome P450 superfamily protein
28	*Ph24ISO2*	Aa05	Peaxi162Scf00695g00218	306.59	4039.95	3.72	Up	delta(24)-sterol reductase
29	CYP87G4	Aa06	Peaxi162Scf00695g00039	4.32	1585.38	8.52	Up	cytochrome P450 superfamily protein
30	CYP88C14	Aa07	Peaxi162Scf00312g00017	891.67	10927.65	3.62	Up	cytochrome P450 superfamily protein
31	*PhCYP88C13.1*	Aa08	Peaxi162Scf00312g00220	7611.31	56159.82	2.88	Up	cytochrome P450 superfamily protein
32	*Ph24ISO1*	Aa09	Peaxi162Scf00312g00221	2014.94	4841.23	1.26	ns	delta(24)-sterol reductase
33	CYP88C15	Aa10	Peaxi162Scf00312g00310	1614.21	5536.27	1.78	Down	cytochrome P450 superfamily protein
34	CYP87G2	Ai01	Peinf101Scf00464g00006	3.07	55.69	4.18	ns	cytochrome P450 superfamily protein
35	CYP87G4	Ai02	Peinf101Scf00464g00009	98.60	3710.03	5.23	Up	cytochrome P450 superfamily protein
36	*Ph24ISO3*	Ai03	Peinf101Scf00464g01010	198.84	2928.94	3.88	Up	delta(24)-sterol reductase
37	CYP88C1	Ai04	Peinf101Scf00464g01009	442.07	7101.50	4.01	Up	cytochrome P450 superfamily protein
38	CYP87G2	Ai05	Peinf101Scf00464g02008	49.37	4712.95	6.58	Up	cytochrome P450 superfamily protein
39	CYP87G2	Ai06	Peinf101Scf00464g02009	117.09	6950.15	5.89	Up	cytochrome P450 superfamily protein
40	CYP88C1	Ai07	Peinf101Scf00464g04011	83.17	2084.82	4.65	Up	cytochrome P450 superfamily protein
41	CYP88C14	Ai08	Peinf101Scf00464g04010	1216.46	11869.43	3.29	Up	cytochrome P450 superfamily protein
42	CYP87G2	Ai09	Peinf101Scf00464g05013	1020.44	28573.65	4.81	ns	cytochrome P450 superfamily protein
43	CYP88C1	Ai10	Peinf101Scf00464g05011	5623.01	20448.03	1.86	Up	cytochrome P450 superfamily protein
44	CYP88C13	Ai11	Peinf101Scf00464g06009	935.88	3167.19	1.76	Up	cytochrome P450 superfamily protein
45	*Ph24ISO4*	Ai12	Peinf101Scf00464g06012	1922.81	5114.29	1.41	Up	delta(24)-sterol reductase
46	CYP88C15	Ai13	Peinf101Scf00464g07005	1189.27	3149.91	1.41	Down	cytochrome P450 superfamily protein
47	AT-II	Ba01	Peaxi162Scf00188g00072	90.99	1186.40	3.70	Up	HXXXD-type acyl-transferase family protein
48	AT-II	Ba02	Peaxi162Scf00188g00713	82.17	1517.90	4.21	Up	HXXXD-type acyl-transferase family protein
49	CYP749B4	Ba03	Peaxi162Scf00188g00718	11.57	1014.72	6.46	Up	cytochrome P450 superfamily protein
50	CYP716A357	Ba04	Peaxi162Scf00188g00719	2.99	163.37	5.77	Up	cytochrome P450 superfamily protein
51	AT-I	Ba05	Peaxi162Scf00188g00081	245.99	1297.17	2.40	Up	HXXXD-type acyl-transferase family protein
52	AT-III	Ba06	Peaxi162Scf00188g00119	189.50	8361.50	5.46	Up	HXXXD-type acyl-transferase family protein
53	DOX-II	Ba07	Peaxi162Scf00188g01224	47.70	344.60	2.85	ns	2-oxoglutarate (2OG) and Fe(II)-dependent oxygenase superfamily protein
54	DOX-I	Ba10	Peaxi162Scf00188g01221	1925.80	10155.36	2.40	Down	2-oxoglutarate (2OG) and Fe(II)-dependent oxygenase superfamily protein
55	CYP88C12	Ba11	Peaxi162Scf00188g01334	12.02	171.36	3.83	ns	cytochrome P450 superfamily protein
56	CYP88C12	Ba12	Peaxi162Scf00188g01335	1620.59	24467.65	3.92	Up	cytochrome P450 superfamily protein
57	other	Ba13	Peaxi162Scf00188g01337	31.69	110.46	1.80	Up	plant basic secretory protein (BSP) family protein
58	AT-I	Ba14	Peaxi162Scf00188g01311	182.76	490.96	1.43	Up	HXXXD-type acyl-transferase family protein
59	AT-I	Ba15	Peaxi162Scf00188g00134	20.00	68.83	1.78	Down	HXXXD-type acyl-transferase family protein
60	AT-II	Bi01	Peinf101Scf01262g02003	33.44	534.98	4.00	Up	HXXXD-type acyl-transferase family protein
61	AT-II	Bi02	Peinf101Scf01262g03011	32.70	619.51	4.24	Up	HXXXD-type acyl-transferase family protein
62	CYP749B4	Bi03	Peinf101Scf01262g03014	25.86	1116.78	5.43	Up	cytochrome P450 superfamily protein
63	AT-I	Bi04	Peinf101Scf01262g03002	243.11	1295.04	2.41	Up	HXXXD-type acyl-transferase family protein
64	other	Bi05	Peinf101Scf01262g04018	498.09	1902.37	1.93	Up	bHLH transcription factor
65	AT-III	Bi06	Peinf101Scf01262g07001	159.72	6189.61	5.28	Up	HXXXD-type acyl-transferase family protein
66	AT-III	Bi07	Peinf101Scf01262g07010	167.90	3664.46	4.45	Up	HXXXD-type acyl-transferase family protein
67	DOX-II	Bi08	Peinf101Scf01262g08014	35.31	289.99	3.04	ns	2-oxoglutarate (2OG) and Fe(II)-dependent oxygenase superfamily protein
68	DOX-I	Bi11	Peinf101Scf01262g09029	41.03	214.43	2.39	ns	2-oxoglutarate (2OG) and Fe(II)-dependent oxygenase superfamily protein
69	DOX-I	Bi12	Peinf101Scf01262g09031	121.38	576.69	2.25	Down	2-oxoglutarate (2OG) and Fe(II)-dependent oxygenase superfamily protein
70	DOX-I	Bi13	Peinf101Scf01262g09032	1824.64	9798.65	2.42	Down	2-oxoglutarate (2OG) and Fe(II)-dependent oxygenase superfamily protein
71	CYP88C12	Bi14	Peinf101Scf01262g09034	2476.44	19386.76	2.97	Up	cytochrome P450 superfamily protein
72	other	Bi15	Peinf101Scf01262g09037	157.97	1066.71	2.76	Up	kirola-like
73	other	Bi16	Peinf101Scf01262g09045	671.10	2683.60	2.00	Up	plant basic secretory protein (BSP) family protein
74	AT-I	Bi17	Peinf101Scf01262g09011	121.28	466.70	1.94	Up	HXXXD-type acyl-transferase family protein
75	CYP749B3	Ca01	Peaxi162Scf01276g00047	526.82	4225.13	3.00	Up	cytochrome P450 superfamily protein
76	AT-III	Ca02	Peaxi162Scf01276g00003	205.25	1764.31	3.10	ns	HXXXD-type acyl-transferase family protein
77	DOX-II	Ca03	Peaxi162Scf01276g00036	4432.93	20227.87	2.19	ns	2-oxoglutarate (2OG) and Fe(II)-dependent oxygenase superfamily protein
78	DOX-II	Ca04	Peaxi162Scf01276g00039	477.24	5767.71	3.60	Up	2-oxoglutarate (2OG) and Fe(II)-dependent oxygenase superfamily protein
79	AT-III	Ci01	Peinf101Scf01278g04015	0.18	9.10	5.68	ns	HXXXD-type acyl-transferase family protein
80	DOX-I	Ci02	Peinf101Scf01278g00010	160.41	3076.03	4.26	ns	2-oxoglutarate (2OG) and Fe(II)-dependent oxygenase superfamily protein
81	AT-I	Ci03	Peinf101Scf01278g00004	67.93	875.94	3.69	Up	HXXXD-type acyl-transferase family protein
82	CYP749B5	Ci04	Peinf101Scf01278g01025	34.15	175.83	2.36	Up	cytochrome P450 superfamily protein
83	CYP749B3	Ci06	Peinf101Scf01278g02017	357.55	3499.34	3.29	Up	cytochrome P450 superfamily protein
84	DOX-II	Ci07	Peinf101Scf01278g03015	4377.61	20142.35	2.20	ns	2-oxoglutarate (2OG) and Fe(II)-dependent oxygenase superfamily protein
85	DOX-II	Ci08	Peinf101Scf01278g03016	475.42	5775.80	3.60	Up	2-oxoglutarate (2OG) and Fe(II)-dependent oxygenase superfamily protein
86	other	Ci09	Peinf101Scf01278g03022	483.39	5068.15	3.39	Up	sulfotransferase
87	other	Ci10	Peinf101Scf01278g04007	571.11	7341.06	3.68	Up	sulfotransferase
88	AT-I	Ci11	Peinf101Scf01278g00005	27.14	67.97	1.32	Down	HXXXD-type acyl-transferase family protein
89	CYP749A370	Ci12	Peinf101Scf01278g04016	85.87	783.66	3.19	ns	cytochrome P450 superfamily protein
90	AT-III	Ci13	Peinf101Scf01278g04005	36.41	316.57	3.12	Down	HXXXD-type acyl-transferase family protein
91	other	Da01	Peaxi162Scf00299g00724	0.66	18.59	4.82	ns	unknown
92	other	Da02	Peaxi162Scf00299g00084	1.30	14.48	3.48	ns	unknown
93	other	Da03	Peaxi162Scf00299g00854	18.44	94.35	2.35	ns	unknown
94	other	Da04	Peaxi162Scf00299g00848	25.90	204.01	2.98	ns	unknown
95	AT-I	Da05	Peaxi162Scf00299g01143	1758.36	3701.11	1.07	Up	HXXXD-type acyl-transferase family protein
96	AT-I	Da06	Peaxi162Scf00299g01145	41.59	145.60	1.81	ns	HXXXD-type acyl-transferase family protein
97	CYP716A356	Da07	Peaxi162Scf00299g01147	21.27	877.32	5.37	Up	cytochrome P450 superfamily protein
98	AT-I	Da08	Peaxi162Scf00299g01113	47.23	679.55	3.85	Up	HXXXD-type acyl-transferase family protein
99	other	Da09	Peaxi162Scf00299g01129	8.75	232.40	4.73	Up	acetyl-CoA-benzylalcohol acetyltransferase
100	AT-I	Da10	Peaxi162Scf00299g01130	101.13	1680.27	4.05	Up	HXXXD-type acyl-transferase family protein

101	AT-I	Di01	Peinf101Scf00791g05032	1724.66	3758.61	1.12	Up	HXXXD-type acyl-transferase family protein
102	CYP716A356	Di02	Peinf101Scf00791g05031	24.85	1125.35	5.50	Up	cytochrome P450 superfamily protein
103	AT-I	Di03	Peinf101Scf00791g05009	18.19	903.63	5.63	Up	HXXXD-type acyl-transferase family protein
104	AT-I	Di04	Peinf101Scf00791g06001	237.67	3464.92	3.87	Up	HXXXD-type acyl-transferase family protein
105	DOX-I	Ea01	Peaxi162Scf00003g04349	328.30	1639.86	2.32	Up	2-oxoglutarate (2OG) and Fe(II)-dependent oxygenase superfamily protein
106	DOX-I	Ea02	Peaxi162Scf00003g04427	56.83	327.20	2.53	ns	2-oxoglutarate (2OG) and Fe(II)-dependent oxygenase superfamily protein
107	DOX-I	Ea03	Peaxi162Scf00003g04426	2476.33	10675.08	2.11	Up	2-oxoglutarate (2OG) and Fe(II)-dependent oxygenase superfamily protein
108	DOX-I	Ea04	Peaxi162Scf00003g04424	140.38	1492.86	3.41	ns	2-oxoglutarate (2OG) and Fe(II)-dependent oxygenase superfamily protein
109	DOX-I	Ea05	Peaxi162Scf00003g05024	0.18	7.37	5.38	Up	2-oxoglutarate (2OG) and Fe(II)-dependent oxygenase superfamily protein
110	DOX-I	Ea06	Peaxi162Scf00003g05025	17.45	107.91	2.63	Up	2-oxoglutarate (2OG) and Fe(II)-dependent oxygenase superfamily protein
111	DOX-I	Ea07	Peaxi162Scf00003g05026	1009.07	6014.64	2.58	Up	2-oxoglutarate (2OG) and Fe(II)-dependent oxygenase superfamily protein
112	other	Ea08	Peaxi162Scf00003g05036	161.25	502.04	1.64	ns	transcription elongation factor (TFIIS) family protein
113	DOX-I	Ea09	Peaxi162Scf00003g05133	137.48	598.48	2.12	Up	2-oxoglutarate (2OG) and Fe(II)-dependent oxygenase superfamily protein
114	AT-III	Fa01	Peaxi162Scf00175g01035	0.18	12.27	6.09	ns	HXXXD-type acyl-transferase family protein
115	DOX-II	Fa02	Peaxi162Scf00175g01030	0.36	101.69	8.16	Up	2-oxoglutarate (2OG) and Fe(II)-dependent oxygenase superfamily protein
116	AT-III	Fa03	Peaxi162Scf00175g00112	84.32	831.38	3.30	ns	HXXXD-type acyl-transferase family protein
117	DOX-I	Fa04	Peaxi162Scf00175g01131	1.32	15.23	3.53	ns	2-oxoglutarate (2OG) and Fe(II)-dependent oxygenase superfamily protein
118	AT-III	Fa05	Peaxi162Scf00175g01139	21.63	153.35	2.83	ns	HXXXD-type acyl-transferase family protein
119	other	Fa06	Peaxi162Scf00175g10117	397.11	2584.86	2.70	Down	sulfotransferase
120	other	Gi01	Peaxi162Scf03271g00006	57.17	620.45	3.44	ns	unknown
121	DOX-I	Gi02	Peaxi162Scf03271g00014	25.43	1360.09	5.74	ns	2-oxoglutarate (2OG) and Fe(II)-dependent oxygenase superfamily protein
122	DOX-I	Gi03	Peaxi162Scf03271g00015	118.85	1567.44	3.72	ns	2-oxoglutarate (2OG) and Fe(II)-dependent oxygenase superfamily protein
123	other	Gi04	Peaxi162Scf03271g00016	802.62	3252.53	2.02	Up	plant basic secretory protein (BSP) family protein
124	AT-I		Peaxi162Scf01060g00110	64.16	1314.44	4.36	Up	HXXXD-type acyl-transferase family protein
125	AT-I		Peaxi162Scf01442g00002	57.04	945.83	4.05	ns	HXXXD-type acyl-transferase family protein
126	AT-I		Peaxi162Scf01732g00002	209.70	546.03	1.38	Up	HXXXD-type acyl-transferase family protein
127	AT-I		Peinf101Scf00822g00007	152.50	393.29	1.37	Up	HXXXD-type acyl-transferase family protein
128	AT-I		Peaxi162Scf00375g00013	46.81	185.46	1.99	ns	HXXXD-type acyl-transferase family protein
129	AT-I		Peinf101Scf01863g00004	27.00	110.27	2.03	ns	HXXXD-type acyl-transferase family protein
130	AT-I		Peinf101Scf04072g01002	20.33	60.91	1.58	ns	HXXXD-type acyl-transferase family protein
131	AT-I		Peaxi162Scf00529g00012	1.33	54.00	5.35	ns	HXXXD-type acyl-transferase family protein
132	AT-I		Peinf101Scf01218g04002	1.32	49.11	5.21	ns	HXXXD-type acyl-transferase family protein
133	AT-I		Peinf101Scf01262g09006	14.33	46.42	1.70	Up	HXXXD-type acyl-transferase family protein
134	AT-I		Peaxi162Scf01142g00002	9.24	36.61	1.99	ns	HXXXD-type acyl-transferase family protein
135	AT-II		Peinf101Scf01262g05004	7.91	117.24	3.89	Up	HXXXD-type acyl-transferase family protein
136	AT-III		Peaxi162Scf00588g00051	72.44	1007.45	3.80	Up	HXXXD-type acyl-transferase family protein
137	CYP87G5P		Peaxi162Scf01218g00211	564.04	42996.07	6.25	Up	cytochrome P450 superfamily protein
138	CYP87G5		Peinf101Scf00498g03007	527.37	41172.60	6.29	Up	cytochrome P450 superfamily protein
139	CYP87G3		Peinf101Scf00289g05004	7.26	607.51	6.39	Up	cytochrome P450 superfamily protein
140	CYP87G3		Peaxi162Scf00586g00077	5.65	436.88	6.27	Up	cytochrome P450 superfamily protein
141	CYP87G4		Peinf101Scf00714g08008	20.89	287.19	3.78	Up	cytochrome P450 superfamily protein
142	CYP88C13		Peinf101Scf00229g04012	84.65	281.61	1.73	ns	cytochrome P450 superfamily protein
143	CYP88C13		Peinf101Scf00229g06005	83.23	249.95	1.59	Up	cytochrome P450 superfamily protein
144	CYP88C13		Peaxi162Scf00002g01923	40.16	131.34	1.71	Up	cytochrome P450 superfamily protein
145	DOX-I		Peaxi162Scf01142g00021	157.06	2626.85	4.06	ns	2-oxoglutarate (2OG) and Fe(II)-dependent oxygenase superfamily protein
146	DOX-I		Peinf101Scf02089g02026	149.11	1627.21	3.45	ns	2-oxoglutarate (2OG) and Fe(II)-dependent oxygenase superfamily protein
147	DOX-I		Peaxi162Scf00003g04123	0.18	99.53	9.09	ns	2-oxoglutarate (2OG) and Fe(II)-dependent oxygenase superfamily protein
148	DOX-I		Peaxi162Scf00538g00615	0.35	77.84	7.78	ns	2-oxoglutarate (2OG) and Fe(II)-dependent oxygenase superfamily protein
149			Peaxi162Scf00120g00019	1371.37	20220.26	3.88	ns	AMP-dependent synthetase and ligase family protein
150			Peinf101Scf00471g06010	3550.84	12632.44	1.83	Up	acetate/butyrate--CoA ligase
151			Peinf101Scf00408g08018	784.53	10468.90	3.74	Up	AMP-dependent synthetase and ligase family protein
152			Peinf101Scf02089g02012	2145.25	9323.75	2.12	Up	2-oxoglutarate (2OG) and Fe(II)-dependent oxygenase superfamily protein
153			Peaxi162Scf00019g00016	2276.00	8637.69	1.92	Up	sterol methyltransferase
154			Peinf101Scf00142g19006	2891.82	7935.82	1.46	Up	ATP sulfurylase
155			Peinf101Scf01294g05042	1395.09	7860.53	2.49	Up	2-oxoglutarate (2OG) and Fe(II)-dependent oxygenase superfamily protein
156			Peaxi162Scf00467g00610	2621.21	7038.17	1.42	Up	sulfate adenylyltransferase
157			Peaxi162Scf00316g00065	1506.81	6111.38	2.02	Up	1,2-dihydroxy-3-keto-5-methylthiopentene dioxygenase
158			Peaxi162Scf00643g00055	2734.96	5936.40	1.12	Up	kunitz trypsin inhibitor
159			Peinf101Scf02093g03001	1377.52	5905.35	2.10	Up	1,2-dihydroxy-3-keto-5-methylthiopentene dioxygenase
160			Peinf101Scf01290g00011	2337.11	5715.72	1.29	Up	glutathione peroxidase
161			Peaxi162Scf00375g00710	2345.99	5695.43	1.28	Up	glutathione peroxidase
162			Peaxi162Scf00089g00933	1097.99	4540.14	2.05	Up	alpha/beta-hydrolases superfamily protein
163			Peaxi162Scf00767g00022	1278.72	4385.94	1.78	Up	unknown
164			Peinf101Scf00506g21016	1156.94	3995.85	1.79	Up	cytochrome b5 protein
165			Peaxi162Scf00382g00132	1766.00	3602.85	1.03	ns	regulator of Vps4 activity in the MVB pathway protein
166			Peinf101Scf02102g00020	1744.07	3584.47	1.04	ns	unknown
167			Peinf101Scf00482g01034	1599.51	3421.33	1.10	Up	miraculin-like
168			Peaxi162Scf00305g00099	630.27	3345.86	2.41	Up	plant basic secretory protein (BSP) family protein
169			Peinf101Scf00252g08004	616.24	3296.86	2.42	Up	unknown
170			Peaxi162Scf00152g01220	1588.89	3264.61	1.04	ns	actin
171			Peinf101Scf00650g23044	1571.12	3259.98	1.05	ns	actin
172			Peinf101Scf02848g00004	1371.45	3116.02	1.18	Up	7-dehydrocholesterol reductase
173			Peaxi162Scf00003g04533	264.02	3111.36	3.56	Up	2-oxoglutarate (2OG) and Fe(II)-dependent oxygenase superfamily protein
174			Peinf101Scf02089g02034	400.90	3074.69	2.94	Up	2-oxoglutarate (2OG) and Fe(II)-dependent oxygenase superfamily protein
175			Peinf101Scf01079g00005	96.35	3047.30	4.98	Up	adenylyl-sulfate kinase
176			Peaxi162Scf00449g00313	96.74	3042.61	4.98	Up	adenylyl-sulfate kinase
177			Peinf101Scf02089g01038	66.01	2543.53	5.27	Up	2-oxoglutarate (2OG) and Fe(II)-dependent oxygenase superfamily protein
178			Peaxi162Scf00089g00833	986.23	2306.11	1.23	Up	alpha/beta-hydrolases superfamily protein
179			Peinf101Scf02107g00002	668.89	2151.25	1.69	Up	unknown
180			Peinf101Scf02107g00022	662.58	2138.03	1.69	Up	unknown
181			Peaxi162Scf00120g00020	785.26	2134.43	1.44	ns	aldehyde dehydrogenase
182			Peinf101Scf01853g05017	50.95	2058.48	5.34	Up	unknown
183			Peaxi162Scf00498g00218	49.86	2040.01	5.35	Up	2-oxoglutarate (2OG) and Fe(II)-dependent oxygenase superfamily protein
184			Peinf101Scf18352g00003	648.57	2025.99	1.64	ns	7-dehydrocholesterol reductase
185			Peaxi162Scf45515g00001	117.58	1768.21	3.91	Up	sulfotransferase
186			Peaxi162Scf22755g00002	586.41	1718.99	1.55	ns	cytochrome P450 superfamily protein
187			Peinf101Scf00482g01004	758.94	1692.69	1.16	Up	kunitz trypsin inhibitor
188			Peinf101Scf00229g08030	491.81	1657.95	1.75	Up	glucuronosyltransferase
189			Peaxi162Scf00525g00018	581.77	1593.69	1.45	ns	3'(2'),5'-bisphosphate nucleotidase
190			Peinf101Scf00408g08019	605.31	1581.85	1.39	ns	aldehyde dehydrogenase
191			Peinf101Scf02778g00040	478.64	1550.65	1.70	Up	60S ribosome subunit biogenesis protein
192			Peaxi162Scf00174g00025	456.12	1499.34	1.72	Up	unknown
193			Peaxi162Scf00016g03455	563.55	1261.46	1.16	ns	oligopeptide transporter
194			Peinf101Scf00679g11007	484.33	1258.77	1.38	ns	SAL1 phosphatase
195			Peinf101Scf00395g04020	557.67	1257.91	1.17	ns	oligopeptide transporter
196			Peaxi162Scf00421g00525	188.37	1233.54	2.71	ns	NRT1/ PTR family protein
197			Peinf101Scf01436g07023	186.29	1223.26	2.72	ns	unknown
198			Peaxi162Scf02065g00021	509.69	1212.82	1.25	ns	nucleotide-diphospho-sugar transferases superfamily protein
199			Peaxi162Scf00366g00066	591.21	1208.74	1.03	ns	unknown
200			Peinf101Scf00144g04018	61.10	1142.72	4.23	ns	unknown

301	Peaxi162Scf00200g00121	85.65	188.32	1.14	ns	unknown
302	Peinf101Scf00427g12014	52.39	187.82	1.84	ns	pathogen-related protein
303	Peinf101Scf00427g13005	49.12	179.95	1.87	ns	pathogen-related protein
304	Peinf101Ctg13044503g0000	26.97	179.41	2.73	ns	acylsugar acyltransferase
305	Peinf101Scf00001g07013	57.24	174.15	1.61	ns	unknown
306	Peinf101Scf00457g08001	8.51	173.09	4.35	Up	UDP-glycosyltransferase
307	Peaxi162Scf00218g00126	64.87	170.65	1.40	ns	eukaryotic aspartyl protease family protein
308	Peinf101Scf00152g11012	2.22	162.24	6.19	ns	clustered mitochondria protein
309	Peinf101Scf16317g00002	79.08	160.38	1.02	ns	unknown
310	Peaxi162Scf00618g00025	72.32	159.81	1.14	Up	P-loop containing nucleoside triphosphate hydrolases superfamily protein
311	Peinf101Scf00898g01002	56.05	149.81	1.42	ns	unknown
312	Peinf101Scf00394g01001	66.20	147.42	1.16	ns	unknown
313	Peinf101Scf02191g01070	3.28	146.37	5.48	Up	epoxide hydrolase
314	Peinf101Scf01175g03028	67.24	143.03	1.09	ns	unknown
315	Peaxi162Scf01003g00017	58.43	142.52	1.29	Up	protein kinase superfamily protein
316	Peinf101Scf00152g07006	50.23	141.68	1.50	Up	NRT1/ PTR family protein
317	Peaxi162Scf01421g00011	35.38	140.99	1.99	ns	polyribonucleotide nucleotidyltransferase
318	Peaxi162Scf00284g00022	35.86	136.52	1.93	ns	peroxidase
319	Peinf101Scf00363g01003	55.18	136.01	1.30	Up	receptor-like kinase
320	Peaxi162Scf00235g00819	63.01	135.19	1.10	Up	membrane bound O-acyl transferase family protein
321	Peaxi162Scf00189g00521	59.23	135.05	1.19	ns	unknown
322	Peinf101Ctg13435204g0000	0.18	134.05	9.53	ns	ABC transporter
323	Peinf101Scf00652g11003	62.55	130.41	1.06	ns	U-box domain-containing protein
324	Peaxi162Scf00183g00021	62.84	130.33	1.05	ns	E3 ubiquitin ligase
325	Peaxi162Scf00207g01313	61.70	129.83	1.07	ns	unknown
326	Peinf101Scf01889g15003	61.42	129.56	1.08	ns	unknown
327	Peaxi162Scf00002g01919	41.78	128.83	1.62	Up	ent-kaurenoic acid oxidase
328	Peaxi162Scf00725g00025	41.72	127.22	1.61	ns	F-box protein
329	Peinf101Scf00605g00030	46.67	125.52	1.43	ns	phosphoenolpyruvate/phosphate translocator
330	Peaxi162Scf00189g00055	16.64	123.89	2.90	Up	UDP-glycosyltransferase
331	Peinf101Scf00384g03002	15.58	123.32	2.98	Up	beta-D-glucosyl crocetin beta-1,6-glucosyltransferase
332	Peaxi162Scf00079g00089	38.06	121.51	1.67	ns	valine--tRNA ligase
333	Peinf101Scf00877g01005	37.87	121.02	1.68	ns	valine--tRNA ligase
334	Peinf101Scf00359g01039	44.56	120.63	1.44	Up	PI-PLC X domain-containing protein
335	Peinf101Scf00973g04011	32.47	120.20	1.89	ns	peroxidase
336	Peinf101Scf00073g05004	55.79	120.05	1.11	ns	unknown
337	Peaxi162Scf00795g00310	51.19	119.53	1.22	ns	leucine-rich repeat protein kinase family protein
338	Peaxi162Scf00366g00711	52.90	115.77	1.13	ns	unknown
339	Peinf101Scf00793g09015	47.39	115.29	1.28	ns	peroxidase
340	Peaxi162Scf00195g00124	47.62	115.25	1.28	ns	peroxidase
341	Peinf101Scf00906g04002	38.97	115.21	1.56	ns	F-box protein
342	Peinf101Scf00791g03029	19.75	114.81	2.54	ns	unknown
343	Peinf101Scf02288g05012	47.38	113.02	1.25	ns	leucine-rich repeat protein kinase family protein
344	Peinf101Scf01822g01034	12.38	109.15	3.14	ns	RNA-binding protein
345	Peaxi162Scf01039g00236	17.91	107.01	2.58	Up	protein kinase superfamily protein
346	Peinf101Scf02318g09035	50.43	103.91	1.04	Up	phosphatidylinositol/phosphatidylcholine transfer protein
347	Peinf101Scf00482g03041	37.48	101.93	1.44	ns	type IV inositol polyphosphate 5-phosphatase
348	Peaxi162Scf00016g00526	49.66	101.25	1.03	Up	Sec14p-like phosphatidylinositol transfer family protein
349	Peaxi162Scf00305g01119	36.90	101.18	1.46	ns	phosphatidylinositol N-acetylglucosaminyltransferase subunit Q
350	Peaxi162Scf00196g00053	49.90	101.07	1.02	ns	xyloglucan-specific endoglucanase inhibitor protein
351	Peinf101Scf03039g06040	41.35	98.57	1.25	ns	unknown
352	Peaxi162Scf00016g03021	41.53	98.25	1.24	ns	unknown
353	Peinf101Scf00216g05027	44.76	94.00	1.07	ns	xyloglucan-specific endoglucanase inhibitor protein
354	Peaxi162Scf00058g00612	36.17	92.39	1.35	Up	NRT1/ PTR family protein
355	Peaxi162Scf00059g00714	44.79	91.87	1.04	ns	WRKY transcription factor
356	Peinf101Ctg13790010g0000	0.00	90.50	22.26	ns	UDP-glycosyltransferase
357	Peinf101Scf00049g03028	5.54	90.09	4.02	Up	2-oxoglutarate (2OG) and Fe(II)-dependent oxygenase superfamily protein
358	Peinf101Scf01822g00011	4.60	89.72	4.28	ns	unknown
359	Peinf101Ctg13307352g0000	0.66	89.72	7.08	Up	acyl-transferase
360	Peinf101Scf00244g01019	31.08	87.86	1.50	ns	laccase
361	Peaxi162Scf00003g03341	39.36	85.32	1.12	Down	Phototropic-responsive NPH3 family protein
362	Peaxi162Scf00304g00620	36.43	83.75	1.20	ns	WRKY transcription factor
363	Peinf101Scf00974g18005	39.32	83.56	1.09	ns	WRKY transcription factor
364	Peinf101Scf00049g12027	37.27	83.54	1.16	ns	NPH3 family protein
365	Peinf101Scf04267g00003	38.94	80.84	1.05	Up	U-box domain-containing protein
366	Peaxi162Scf00376g00324	23.00	77.98	1.76	ns	mediator of RNA polymerase II transcription subunit 23
367	Peaxi162Scf00744g00337	13.08	75.64	2.53	ns	receptor-like kinase
368	Peaxi162Scf00502g00011	16.37	75.56	2.21	Up	UDP-glycosyltransferase
369	Peinf101Scf04325g00003	36.62	75.38	1.04	ns	histone-lysine N-methyltransferase,
370	Peinf101Scf00140g27001	16.28	74.47	2.19	Up	UDP-glycosyltransferase
371	Peinf101Scf01822g00007	7.58	72.84	3.26	ns	unknown
372	Peaxi162Scf00001g10028	28.95	71.38	1.30	Up	seed storage 2S albumin superfamily protein
373	Peinf101Scf04653g00001	23.99	71.19	1.57	ns	NAD kinase
374	Peinf101Scf00339g01023	30.59	70.75	1.21	ns	WRKY transcription factor
375	Peaxi162Scf00001g00373	32.07	70.27	1.13	ns	pectin methylesterase inhibitor superfamily
376	Peinf101Scf00791g11026	26.51	69.32	1.39	Up	seed storage 2S albumin superfamily protein
377	Peaxi162Scf01149g00114	20.81	67.63	1.70	ns	laccase
378	Peinf101Scf02760g00025	32.69	67.51	1.05	Down	acyl-CoA N-acyltransferases (NAT) superfamily protein
379	Peinf101Scf00049g00023	19.35	66.17	1.77	ns	2-oxoglutarate (2OG) and Fe(II)-dependent oxygenase superfamily protein
380	Peinf101Scf00039g17003	18.48	64.66	1.81	Up	unknown
381	Peinf101Scf02496g00046	7.25	62.92	3.12	ns	protein kinase superfamily protein
382	Peaxi162Scf00135g01433	13.90	62.64	2.17	ns	Integrase-type DNA-binding superfamily protein
383	Peaxi162Scf00423g00611	28.75	61.84	1.10	ns	UDP-glycosyltransferase
384	Peaxi162Scf00166g00337	21.84	60.39	1.47	Up	PLC-like phosphodiesterases superfamily protein
385	Peaxi162Scf00230g00068	28.54	59.37	1.06	ns	unknown
386	Peinf101Scf00487g17001	18.01	58.67	1.70	ns	major facilitator superfamily protein
387	Peinf101Ctg13609722g0000	0.18	57.47	8.31	ns	glutamate-1-semialdehyde 2,1-aminomutase 2
388	Peaxi162Scf00493g00120	24.21	57.40	1.25	ns	unknown
389	Peinf101Scf07836g00008	0.18	54.02	8.22	ns	unknown
390	Peinf101Scf03371g00019	0.99	53.91	5.77	Up	alpha/beta-hydrolases superfamily protein
391	Peinf101Scf00133g01014	6.59	53.31	3.02	ns	seed storage 2S albumin superfamily protein
392	Peaxi162Scf01217g00013	4.56	51.11	3.49	ns	unknown
393	Peaxi162Scf00311g01311	15.78	50.16	1.67	ns	major facilitator superfamily protein
394	Peaxi162Scf00366g00216	15.43	49.75	1.69	ns	alpha/beta-hydrolases superfamily protein
395	Peaxi162Scf00909g00026	10.97	48.71	2.15	ns	unknown
396	Peaxi162Scf00128g01137	18.66	46.92	1.33	ns	leucine-rich repeat protein kinase family protein
397	Peaxi162Scf01828g00001	1.64	42.64	4.70	ns	HXXXD-type acyl-transferase family protein
398	Peaxi162Scf00815g00116	4.59	42.26	3.20	ns	unknown
399	Peinf101Scf04549g00005	8.95	40.88	2.19	ns	protein kinase superfamily protein
400	Peinf101Scf00782g11019	6.88	40.58	2.56	ns	receptor-like kinase

401	Peaxi162Scf00241g00628	3.32	40.32	3.60	Up	heat shock protein
402	Peaxi162Scf00002g02012	11.05	39.74	1.85	Up	unknown
403	Peinf101Scf00408g14003	0.66	39.46	5.90	ns	HXXXD-type acyl-transferase family protein
404	Peinf101Scf01126g00005	12.37	37.05	1.58	ns	tetraspanin family protein
405	Peaxi162Scf00236g00216	12.42	36.92	1.57	ns	tetraspanin family protein
406	Peinf101Scf00734g00028	5.95	36.66	2.62	ns	calcium-dependent lipid-binding family protein
407	Peinf101Scf01349g05007	6.99	35.50	2.34	ns	defensin-like protein
408	Peaxi162Scf00324g00722	7.03	35.38	2.33	ns	defensin-like protein
409	Peinf101Scf01614g01042	2.61	35.01	3.74	ns	HXXXD-type acyl-transferase family protein
410	Peinf101Scf00665g03007	0.00	34.34	20.91	ns	epidermis-specific secreted glycoprotein
411	Peinf101Scf00142g03009	3.26	34.22	3.39	ns	seed storage 2S albumin superfamily protein
412	Peaxi162Scf00402g00513	3.28	34.11	3.38	ns	seed storage 2S albumin superfamily protein
413	Peaxi162Scf00196g00119	1.98	34.11	4.11	ns	unknown
414	Peinf101Scf01427g07020	10.14	33.85	1.74	ns	alpha/beta-hydrolases superfamily protein
415	Peinf101Scf00408g07016	1.63	33.77	4.37	ns	AMP-dependent synthetase and ligase family protein
416	Peinf101Scf09393g00003	8.49	33.40	1.98	ns	unknown
417	Peinf101Scf00832g09005	7.59	30.16	1.99	ns	peroxidase
418	Peaxi162Scf00690g00031	8.57	29.35	1.78	Up	RING/U-box superfamily protein
419	Peinf101Scf01767g01006	3.61	28.91	3.00	ns	seed storage 2S albumin superfamily protein
420	Peaxi162Scf01158g00313	3.93	28.58	2.86	ns	seed storage 2S albumin superfamily protein
421	Peaxi162Scf00018g00194	9.28	28.56	1.62	ns	unknown
422	Peinf101Scf10568g00011	1.64	28.18	4.10	Up	heat shock protein
423	Peaxi162Scf00877g00009	7.23	26.68	1.88	ns	unknown
424	Peaxi162Scf00516g00216	4.97	26.59	2.42	ns	HXXXD-type acyl-transferase family protein
425	Peinf101Scf10105g00003	3.96	26.21	2.73	ns	unknown
426	Peinf101Scf01099g02023	3.62	25.30	2.81	ns	unknown
427	Peinf101Scf01441g01014	6.61	24.74	1.91	ns	conserved hypothetical protein
428	Peaxi162Scf01217g00110	1.95	23.83	3.61	ns	unknown
429	Peaxi162Scf00258g00617	0.18	23.65	7.03	ns	2-oxoglutarate (2OG) and Fe(II)-dependent oxygenase superfamily protein
430	Peaxi162Scf00000g02515	5.98	21.61	1.85	ns	actin
431	Peinf101Scf00061g01020	3.30	20.96	2.67	ns	G-type lectin S-receptor-like serine/threonine-protein kinase
432	Peinf101Scf00889g10041	0.35	20.83	5.88	ns	lysine histidine transporter
433	Peaxi162Scf00783g00133	2.63	19.50	2.89	ns	seed storage 2S albumin superfamily protein
434	Peinf101Scf16478g00005	0.67	18.45	4.79	ns	quinone oxidoreductase-like protein
435	Peaxi162Scf00045g00325	1.63	17.85	3.45	ns	bZIP transcription factor
436	Peinf101Scf00191g29018	2.95	17.67	2.58	ns	pectin lyase-like superfamily protein
437	Peaxi162Scf00052g01011	2.96	17.61	2.57	ns	pectin lyase-like superfamily protein
438	Peaxi162Scf00058g00010	2.28	15.65	2.78	ns	NRT1/ PTR family protein
439	Peaxi162Scf00039g01626	0.35	13.10	5.21	Up	NAD(P)-binding Rossmann-fold superfamily protein
440	Peinf101Ctg13196654g0000	0.18	11.81	6.05	ns	unknown
441	Peinf101Scf05594g00003	0.18	11.70	6.03	ns	unknown
442	Peinf101Ctg13554698g0000	0.18	9.91	5.79	ns	unknown
443	Peaxi162Scf00408g00522	0.18	9.13	5.67	ns	unknown
444	Peaxi162Scf00982g00214	0.18	8.68	5.60	ns	limit dextrinase
445	Peinf101Scf05146g00020	0.18	8.22	5.53	Up	acyl-activating enzyme

Note: Gene models IDs of *Petunia axillaris* are colored in green. FC; fold change, ns; not significant.

Table S3 Expression data of PhERF1-downregulated genes

				PhERF1 overexpression (OX) experiment			
				Normalized expression values			
No.	Gene name or family	Label	Gene model ID	Control	*PhERF1* OX	Log_2FC	Description
1	*PhHMGR4*		Peaxi162Scf01393g00015	2751.66	1295.04	-1.09	3-hydroxy-3-methylglutaryl-coenzyme A reductase
2	*PhHMGR5*		Peinf101Scf00393g02016	2740.02	1299.98	-1.08	3-hydroxy-3-methylglutaryl-coenzyme A reductase
3	CYP749B6	Ba08	Peaxi162Scf00188g01219	2635.75	1072.35	-1.30	cytochrome P450 superfamily protein
4	CYP749B6	Ba09	Peaxi162Scf00188g01225	2459.21	673.99	-1.87	cytochrome P450 superfamily protein
5	CYP749B6	Bi09	Peinf101Scf01262g08002	3464.33	1199.49	-1.53	cytochrome P450 superfamily protein
6	CYP749B6	Bi10	Peinf101Scf01262g08023	1377.75	470.98	-1.55	cytochrome P450 superfamily protein
7	CYP749B3	Ci05	Peinf101Scf01278g02001	45.05	0.00	-22.02	cytochrome P450 superfamily protein
8			Peinf101Scf00536g11008	95070.89	35691.85	-1.41	cysteine proteinases superfamily protein
9			Peaxi162Scf00357g00012	53337.97	16121.62	-1.73	terpene synthase
10			Peinf101Scf00805g01027	47504.30	23339.05	-1.03	endochitinase A
11			Peinf101Scf01168g02024	44413.24	13975.67	-1.67	terpene synthase
12			Peinf101Scf01455g00010	43160.66	12963.64	-1.74	cytochrome P450 superfamily protein
13			Peaxi162Scf00875g00019	41495.73	20112.66	-1.04	endochitinase A
14			Peaxi162Scf00032g00106	40333.27	11497.34	-1.81	glucan endo-1,3-beta-glucosidase
15			Peaxi162Scf00439g00038	25785.30	12033.26	-1.10	cytochrome P450 superfamily protein
16			Peinf101Scf00562g02015	25601.91	12039.64	-1.09	cytochrome P450 superfamily protein
17			Peaxi162Scf00282g00713	24260.13	7438.95	-1.71	cysteine proteinases superfamily protein
18			Peaxi162Scf01118g00004	24260.13	7438.95	-1.71	cysteine proteinases superfamily protein
19			Peinf101Scf03539g00020	23653.59	6464.38	-1.87	allene oxide synthase
20			Peaxi162Scf00228g00020	20202.48	8563.90	-1.24	glucan endo-1,3-beta-glucosidase
21			Peaxi162Scf00071g00319	18670.97	2643.94	-2.82	terpene synthase
22			Peaxi162Scf00409g00216	16198.59	7252.50	-1.16	terpene synthase
23			Peaxi162Scf00016g01221	16126.17	4201.58	-1.94	allene oxide synthase
24			Peinf101Scf00650g16030	15628.00	2147.07	-2.86	terpene synthase
25			Peinf101Scf02809g02002	14567.85	6326.88	-1.20	glucan endo-1,3-beta-glucosidase
26			Peinf101Scf00564g03027	12200.03	5542.49	-1.14	terpene synthase
27			Peinf101Scf00962g03033	12014.31	5463.10	-1.14	pathogenesis-related thaumatin superfamily protein
28			Peaxi162Scf00714g00213	11503.85	5242.43	-1.13	proteinase inhibitor I
29			Peaxi162Scf00016g01228	10932.93	2902.85	-1.91	allene oxide synthase
30			Peaxi162Scf00251g00081	10862.06	5214.43	-1.06	non-specific lipid-transfer protein
31			Peinf101Scf01455g00001	10712.80	3229.73	-1.73	glucan endo-1,3-beta-glucosidase
32			Peaxi162Scf00045g01022	10098.53	3420.77	-1.56	cis-prenyl transferase
33			Peaxi162Scf00008g00441	9988.49	3979.05	-1.33	UDP-glycosyltransferase superfamily protein
34			Peinf101Scf00930g02010	9922.16	3986.28	-1.32	UDP-glycosyltransferase superfamily protein
35			Peinf101Scf00146g09014	6134.85	2057.07	-1.58	cis-prenyl transferase
36			Peaxi162Scf00152g00067	6047.36	332.13	-4.19	CAP (Cysteine-rich secretory proteins, Antigen 5, and Pathogenesis-related 1 protein) superfamily protein
37			Peinf101Scf03777g00014	5101.44	1364.96	-1.90	allene oxide synthase
38			Peaxi162Scf00030g01110	4757.06	1282.88	-1.89	UDP-glycosyltransferase superfamily protein
39			Peinf101Scf00736g17005	4746.22	2190.99	-1.12	branched-chain-amino-acid aminotransferase
40			Peinf101Scf00650g29007	4728.23	252.37	-4.23	CAP (Cysteine-rich secretory proteins, Antigen 5, and Pathogenesis-related 1 protein) superfamily protein
41			Peaxi162Scf00232g01124	4460.91	1429.90	-1.64	cis-prenyl transferase
42			Peinf101Scf00007g09010	4457.94	1227.03	-1.86	UDP-glycosyltransferase superfamily protein
43			Peaxi162Scf01254g00022	4395.58	1835.14	-1.26	BAG family molecular chaperone regulator
44			Peaxi162Scf01566g00007	4333.41	823.59	-2.40	cytochrome P450 superfamily protein
45			Peaxi162Scf00193g00117	4276.48	873.45	-2.29	terpene synthase
46			Peaxi162Scf00102g00119	4257.07	1091.11	-1.96	NRT1/ PTR FAMILY protein
47			Peaxi162Scf00305g00091	3981.22	1518.15	-1.39	NAD(P)-binding Rossmann-fold superfamily protein
48			Peinf101Scf00252g06004	3960.07	1522.56	-1.38	NAD(P)-binding Rossmann-fold superfamily protein
49			Peinf101Scf00545g01028	3825.21	756.16	-2.34	terpene synthase
50			Peinf101Scf00564g02018	3769.15	1686.62	-1.16	terpene synthase
51			Peaxi162Scf00628g00019	3651.27	1740.24	-1.07	branched-chain-amino-acid aminotransferase
52			Peaxi162Scf00500g00034	3470.47	1536.24	-1.18	unknown
53			Peaxi162Scf00366g00819	3412.15	1167.93	-1.55	phosphatidic acid phosphatase (PAP2) family protein
54			Peinf101Scf02382g09059	3393.07	862.65	-1.98	NRT1/ PTR FAMILY protein
55			Peinf101Scf01061g02010	3255.17	1484.04	-1.13	glutathione S-transferase
56			Peaxi162Scf00384g00008	3026.12	1107.71	-1.45	cytochrome P450 superfamily protein
57			Peinf101Scf01568g05013	2991.16	1102.36	-1.44	cytochrome P450 superfamily protein
58			Peinf101Scf00073g06023	2823.31	982.71	-1.52	phosphatidic acid phosphatase (PAP2) family protein
59			Peaxi162Scf00300g00848	2742.77	1160.27	-1.24	subtilase family protein
60			Peinf101Scf01539g03001	2660.54	1168.22	-1.19	unknown
61			Peaxi162Scf00610g00022	2439.28	881.50	-1.47	cytochrome P450 superfamily protein
62			Peaxi162Scf00399g00920	2430.39	1069.19	-1.18	glutathione S-transferase
63			Peinf101Scf00962g04031	2382.80	338.38	-2.82	pathogenesis-related thaumatin superfamily protein
64			Peaxi162Scf00206g00078	2120.38	1015.69	-1.06	RING membrane-anchor
65			Peaxi162Scf00113g00024	2094.57	294.59	-2.83	cytochrome P450 superfamily protein
66			Peaxi162Scf00610g00021	2084.67	695.34	-1.58	cytochrome P450 superfamily protein
67			Peaxi162Scf00016g00332	1962.58	923.30	-1.09	CAP (Cysteine-rich secretory proteins, Antigen 5, and Pathogenesis-related 1 protein) superfamily protein
68			Peinf101Scf00278g04020	1957.17	819.08	-1.26	BAG family molecular chaperone regulator
69			Peinf101Scf02382g09065	1946.40	449.05	-2.12	NRT1/ PTR FAMILY protein
70			Peinf101Scf00395g05049	1890.82	909.63	-1.06	CAP (Cysteine-rich secretory proteins, Antigen 5, and Pathogenesis-related 1 protein) superfamily protein
71			Peinf101Scf00199g02005	1801.52	254.09	-2.83	cytochrome P450 superfamily protein
72			Peinf101Scf00168g16001	1765.30	841.79	-1.07	RING membrane-anchor
73			Peaxi162Scf00075g01548	1726.59	593.83	-1.54	UDP-glycosyltransferase superfamily protein
74			Peinf101Scf05044g00002	1705.95	782.51	-1.12	unknown
75			Peaxi162Scf00415g00216	1680.29	780.39	-1.11	long-chain-alcohol oxidase FAO
76			Peaxi162Scf00218g00073	1644.63	637.48	-1.37	FAD-binding Berberine family protein
77			Peinf101Scf00670g04002	1636.18	638.28	-1.36	FAD-binding Berberine family protein
78			Peaxi162Scf00110g00123	1622.60	790.14	-1.04	CHY-type/CTCHY-type/RING-type Zinc finger protein
79			Peinf101Scf01317g00028	1620.37	566.41	-1.52	UDP-glycosyltransferase superfamily protein
80			Peaxi162Scf00002g02718	1599.74	636.41	-1.33	heat stress transcription factor
81			Peaxi162Scf00724g00072	1535.86	239.96	-2.68	HXXXD-type acyl-transferase family protein
82			Peinf101Scf00823g05003	1524.29	240.11	-2.67	HXXXD-type acyl-transferase family protein
83			Peaxi162Scf00602g00008	1461.90	420.94	-1.80	Long-chain-alcohol oxidase FAO
84			Peaxi162Scf00165g00818	1347.98	595.02	-1.18	haloacid dehalogenase-like hydrolase (HAD) superfamily protein
85			Peinf101Scf00985g07015	1336.20	593.94	-1.17	haloacid dehalogenase-like hydrolase (HAD) superfamily protein
86			Peaxi162Scf00264g00214	1288.11	530.16	-1.28	peroxidase
87			Peinf101Scf00138g01013	1276.34	528.28	-1.27	peroxidase
88			Peinf101Scf00879g10015	1248.69	593.65	-1.07	long-chain-alcohol oxidase FAO
89			Peinf101Scf03539g00043	1220.78	209.08	-2.55	allene oxide synthase
90			Peaxi162Scf00007g00012	1184.56	451.36	-1.39	HXXXD-type acyl-transferase family protein
91			Peaxi162Scf00102g01137	1170.21	239.87	-2.29	NRT1/ PTR FAMILY protein
92			Peinf101Scf00805g05014	1086.37	496.19	-1.13	lysine histidine transporter
93			Peinf101Scf00151g10020	1046.04	380.91	-1.46	Leucine-rich receptor-like protein kinase family protein
94			Peinf101Scf02030g08041	1045.39	458.88	-1.19	purple acid phosphatase
95			Peaxi162Scf00189g00003	1034.78	504.06	-1.04	monothiol glutaredoxin-S5
96			Peaxi162Scf00220g00118	1029.47	461.50	-1.16	somatic embryogenesis receptor-like kinase
97			Peinf101Scf01048g02004	1016.77	409.98	-1.31	heat stress transcription factor
98			Peaxi162Scf00301g00312	1003.29	473.71	-1.08	expansin
99			Peinf101Scf00876g10009	991.11	472.33	-1.07	expansin
100			Peaxi162Scf00875g00228	985.56	401.79	-1.29	lysine histidine transporter

es

201	Peaxi162Scf00382g00033	143.88	47.58	-1.60	unknown
202	Peinf101Scf02263g00003	130.00	43.45	-1.58	unknown
203	Peinf101Scf00169g23008	123.24	39.38	-1.65	pistil extensin like protein
204	Peaxi162Scf00815g00018	122.50	33.37	-1.88	2-oxoglutarate (2OG) and Fe(II)-dependent oxygenase superfamily protein
205	Peinf101Scf00059g13006	120.82	0.18	-9.36	polyribonucleotide nucleotidyltransferase, putative
206	Peinf101Scf01139g05007	120.61	42.57	-1.50	NOD26-like intrinsic protein
207	Peinf101Scf00073g11021	117.39	51.41	-1.19	peroxidase
208	Peinf101Scf04519g01027	116.89	31.41	-1.90	2-oxoglutarate (2OG) and Fe(II)-dependent oxygenase superfamily protein
209	Peinf101Scf00973g06041	107.72	45.90	-1.23	receptor like protein
210	Peinf101Scf14618g00002	104.99	21.45	-2.29	glucan endo-1,3-beta-glucosidase
211	Peinf101Scf04519g02015	104.67	16.00	-2.71	hydrogen peroxide-induced protein
212	Peinf101Scf02360g01061	103.75	25.96	-2.00	alpha dioxygenase
213	Peinf101Scf01355g02014	102.42	11.38	-3.17	terpene synthase
214	Peaxi162Scf00451g00323	101.98	19.85	-2.36	glucan endo-1,3-beta-glucosidase
215	Peaxi162Scf00129g00942	101.63	27.14	-1.90	alpha dioxygenase
216	Peaxi162Scf00026g00305	101.45	48.62	-1.06	HXXXD-type acyl-transferase family protein
217	Peaxi162Scf00140g00066	100.21	33.97	-1.56	P-loop containing nucleoside triphosphate hydrolases superfamily protein
218	Peinf101Scf00116g06022	99.85	31.90	-1.65	unknown
219	Peaxi162Scf00415g00023	99.57	40.78	-1.29	Long-chain-alcohol oxidase FAO
220	Peaxi162Scf00791g00023	99.57	39.47	-1.34	ARM repeat superfamily protein
221	Peinf101Scf01192g04049	99.18	19.79	-2.32	WRKY DNA-binding protein
222	Peaxi162Scf00406g01119	97.54	30.22	-1.69	unknown
223	Peinf101Scf01214g04044	96.41	18.23	-2.40	glucan endo-1,3-beta-glucosidase
224	Peinf101Ctg13727862g00001	91.31	32.17	-1.51	unknown
225	Peaxi162Scf00064g00002	90.02	31.76	-1.50	unknown
226	Peaxi162Scf00965g00001	88.40	23.92	-1.89	cytochrome P450 superfamily protein
227	Peinf101Scf00650g25029	85.97	15.92	-2.43	LL-diaminopimelate aminotransferase
228	Peaxi162Scf00086g00054	85.89	37.67	-1.19	receptor like protein
229	Peinf101Scf00028g05011	84.64	20.47	-2.05	alpha/beta-Hydrolases superfamily protein
230	Peaxi162Scf00152g01120	84.24	15.55	-2.44	LL-diaminopimelate aminotransferase
231	Peaxi162Scf01644g00001	81.96	26.81	-1.61	cytochrome P450 superfamily protein
232	Peinf101Scf00750g06042	81.89	39.28	-1.06	peroxidase
233	Peaxi162Scf00091g00176	81.57	27.54	-1.57	cytochrome P450 superfamily protein
234	Peaxi162Scf01054g00024	80.95	37.20	-1.12	peroxidase
235	Peinf101Scf05124g00012	80.84	24.13	-1.74	ATPase ASNA1 homolog
236	Peaxi162Scf00062g01613	80.57	18.05	-2.16	alpha/beta-Hydrolases superfamily protein
237	Peinf101Scf02360g01060	80.49	20.50	-1.97	alpha dioxygenase
238	Peinf101Scf02550g00009	79.77	38.65	-1.05	bifunctional aspartokinase/homoserine dehydrogenase
239	Peinf101Scf01748g00018	78.92	0.00	-22.78	methionine aminopeptidase
240	Peaxi162Scf00089g01132	78.34	21.50	-1.87	Indole-3-glycerol phosphate synthase
241	Peinf101Scf02393g02016	78.05	29.81	-1.39	P-loop containing nucleoside triphosphate hydrolases superfamily protein
242	Peinf101Scf01093g02011	77.73	25.86	-1.59	cytochrome P450 superfamily protein
243	Peaxi162Scf00023g00001	76.84	9.36	-3.04	terpene synthase
244	Peaxi162Scf00561g00021	76.73	22.73	-1.76	L-tyrosine decarboxylase
245	Peaxi162Scf00574g00118	76.69	36.29	-1.08	NRT1/ PTR FAMILY protein
246	Peinf101Scf01626g05013	76.39	36.42	-1.07	NRT1/ PTR FAMILY protein
247	Peinf101Scf01192g04045	74.10	28.17	-1.40	peroxidase
248	Peaxi162Scf00420g00547	74.04	28.07	-1.40	peroxidase
249	Peaxi162Scf00316g00059	73.01	30.13	-1.28	early light-induced protein
250	Peaxi162Scf00658g00426	72.85	28.86	-1.34	regulator of chromosome condensation (RCC1) family protein
251	Peinf101Ctg13714623g00003	70.35	0.00	-22.62	unknown
252	Peaxi162Scf00004g03921	69.29	12.58	-2.46	DNA binding,zinc ion binding,DNA binding
253	Peaxi162Scf00050g00138	68.07	20.89	-1.70	xyloglucan-specific endoglucanase inhibitor 11 [Solanum tuberosum]
254	Peinf101Scf00032g08009	66.21	18.24	-1.86	unknown
255	Peinf101Scf01016g08021	62.39	23.57	-1.40	disease resistance family protein / LRR family protein
256	Peaxi162Scf00304g00719	62.27	28.02	-1.15	WRKY DNA-binding protein
257	Peinf101Scf02661g01013	58.61	21.63	-1.44	aspartyl protease family protein
258	Peinf101Scf00339g02023	58.20	26.04	-1.16	WRKY DNA-binding protein
259	Peinf101Scf00007g01012	57.68	23.50	-1.30	alcohol dehydrogenase
260	Peaxi162Scf00196g00613	57.39	15.28	-1.91	xyloglucan-specific endoglucanase inhibitor protein 2 [Solanum tuberosum]
261	Peinf101Scf00985g02002	56.54	17.19	-1.72	L-tyrosine decarboxylase
262	Peinf101Scf00530g01012	56.13	22.62	-1.31	respiratory burst oxidase protein F
263	Peaxi162Scf00027g00177	54.12	13.25	-2.03	unknown
264	Peaxi162Scf00418g00114	53.77	19.96	-1.43	respiratory burst oxidase protein F
265	Peinf101Scf00962g19026	52.71	17.27	-1.61	cation/H(+) antiporter
266	Peaxi162Scf00420g00640	52.15	9.42	-2.47	WRKY DNA-binding protein
267	Peaxi162Scf00803g00412	49.01	9.74	-2.33	unknown
268	Peinf101Scf00011g03016	48.67	11.25	-2.11	unknown
269	Peaxi162Scf00003g00168	48.28	19.12	-1.34	alpha/beta-Hydrolases superfamily protein
270	Peinf101Scf00498g08022	48.14	12.60	-1.93	cytochrome P450 superfamily protein
271	Peinf101Scf00914g00018	46.61	19.64	-1.25	germin-like protein
272	Peaxi162Scf00258g00719	45.11	16.22	-1.48	cysteine/histidine-rich C1 domain family protein
273	Peaxi162Scf00171g01120	44.64	16.98	-1.39	bHLH DNA-binding superfamily protein
274	Peaxi162Scf01000g00115	44.49	17.23	-1.37	germin-like protein
275	Peaxi162Scf00690g00052	43.81	14.02	-1.64	ERF transcription factor
276	Peinf101Scf00027g04002	43.64	13.74	-1.67	ERF transcription factor
277	Peaxi162Scf00534g00012	40.48	8.40	-2.27	flowering promoting factor 1
278	Peinf101Scf04561g00055	38.34	0.00	-21.80	magnesium transporter NIPA2
279	Peaxi162Scf00009g00081	36.30	0.18	-7.63	disease resistance protein (CC-NBS-LRR class) family
280	Peaxi162Scf00993g00339	36.20	9.99	-1.86	mitogen-activated protein kinase kinase kinase
281	Peaxi162Scf00046g00818	34.75	4.30	-3.01	tetratricopeptide repeat (TPR)-like superfamily protein
282	Peinf101Scf00713g11022	34.68	10.02	-1.79	mitogen-activated protein kinase kinase kinase
283	Peaxi162Scf00238g00062	34.60	4.01	-3.11	plant invertase/pectin methylesterase inhibitor superfamily protein
284	Peinf101Scf00174g00001	34.13	4.03	-3.08	plant invertase/pectin methylesterase inhibitor superfamily protein
285	Peinf101Scf01113g00005	33.11	6.01	-2.46	flowering promoting factor 1
286	Peaxi162Scf00101g00910	27.56	7.32	-1.91	RmlC-like cupins superfamily protein
287	Peinf101Scf01468g09048	27.31	0.18	-7.22	brefeldin A-inhibited guanine nucleotide-exchange protein
288	Peaxi162Scf00334g00210	26.04	2.43	-3.42	unknown
289	Peinf101Scf04208g01005	24.29	5.67	-2.10	adenosine 3-phospho 5-phosphosulfate transporter
290	Peaxi162Scf00075g00121	21.32	1.32	-4.01	terpene synthase
291	Peinf101Scf00252g01009	20.23	0.18	-6.79	unknown
292	Peinf101Scf02030g01007	19.36	1.98	-3.29	tetratricopeptide repeat (TPR)-like superfamily protein
293	Peinf101Scf00032g06012	16.38	0.18	-6.49	unknown
294	Peinf101Scf02107g02073	16.17	1.68	-3.27	cytochrome P450 superfamily protein
295	Peinf101Scf03767g00004	9.62	0.18	-5.73	cytochrome P450 superfamily protein
296	Peaxi162Scf01013g00225	7.67	0.18	-5.42	polynucleotide adenylyltransferase family protein

Note: Gene models IDs of Petunia axillaris are colored in green. FC; fold change.

Table S4 Gene Ontology (GO) terms enriched in differentially expressed genes (DEGs) up- or down-regulated by PhERF1

GO ID	GO Term	Count	*p*-value	Aspect
P. axillaris DEGs up-regulated by PhERF1				
GO:0055114	oxidation-reduction process	61	2.20E-20	P
GO:0044710	single-organism metabolic process	72	3.40E-11	P
GO:0016126	sterol biosynthetic process	5	1.50E-09	P
GO:0006694	steroid biosynthetic process	7	1.60E-08	P
GO:0008202	steroid metabolic process	7	1.40E-07	P
GO:0016125	sterol metabolic process	5	2.00E-07	P
GO:0046165	alcohol biosynthetic process	7	2.70E-07	P
GO:1901617	organic hydroxy compound biosynthetic process	7	1.20E-05	P
GO:0006066	alcohol metabolic process	7	5.20E-05	P
GO:0008610	lipid biosynthetic process	11	0.00065	P
GO:1901615	organic hydroxy compound metabolic process	7	0.00069	P
GO:0016491	oxidoreductase activity	63	3.10E-22	F
GO:0003824	catalytic activity	127	3.70E-18	F
GO:0016747	transferase activity, transferring acyl groups other than amino-acyl groups	25	4.00E-15	F
GO:0016746	transferase activity, transferring acyl groups	25	4.40E-14	F
GO:0016705	oxidoreductase activity, acting on paired donors, with incorporation or reduction of molecular oxygen	24	1.30E-10	F
GO:0005506	iron ion binding	24	1.70E-10	F
GO:0020037	heme binding	23	2.10E-08	F
GO:0046906	tetrapyrrole binding	23	2.60E-08	F
GO:0016740	transferase activity	44	0.00078	F
GO:0071944	cell periphery	17	0.00011	C
P. inflata DEGs up-regulated by PhERF1				
GO:0055114	oxidation-reduction process	56	6.30E-16	P
GO:0044710	single-organism metabolic process	71	3.30E-10	P
GO:0006694	steroid biosynthetic process	8	1.10E-09	P
GO:0016126	sterol biosynthetic process	5	6.00E-09	P
GO:0008202	steroid metabolic process	8	1.10E-08	P
GO:0046165	alcohol biosynthetic process	8	1.30E-08	P
GO:0016125	sterol metabolic process	5	2.80E-07	P
GO:1901617	organic hydroxy compound biosynthetic process	8	1.30E-06	P
GO:0006066	alcohol metabolic process	8	4.20E-06	P
GO:1901615	organic hydroxy compound metabolic process	8	9.20E-05	P
GO:0044699	single-organism process	89	9.60E-05	P
GO:0016129	phytosteroid biosynthetic process	3	0.00014	P
GO:0016132	brassinosteroid biosynthetic process	3	0.00014	P
GO:0016128	phytosteroid metabolic process	3	0.00023	P
GO:0016131	brassinosteroid metabolic process	3	0.00023	P
GO:0008610	lipid biosynthetic process	11	0.00044	P
GO:0016491	oxidoreductase activity	58	1.30E-18	F
GO:0003824	catalytic activity	126	2.20E-18	F
GO:0016747	transferase activity, transferring acyl groups other than amino-acyl groups	24	1.40E-14	F
GO:0016746	transferase activity, transferring acyl groups	24	1.10E-13	F
GO:0016705	oxidoreductase activity, acting on paired donors, with incorporation or reduction of molecular oxygen	27	3.20E-13	F
GO:0005506	iron ion binding	26	4.40E-12	F
GO:0020037	heme binding	27	3.90E-11	F
GO:0046906	tetrapyrrole binding	27	5.60E-11	F
GO:0000254	C-4 methylsterol oxidase activity	2	5.70E-05	F
GO:0016740	transferase activity	47	8.20E-05	F
P. axillaris DEGs down-regulated by PhERF1				
GO:0055114	oxidation-reduction process	30	7.50E-06	P
GO:0002215	defense response to nematode	2	5.00E-05	P
GO:0009814	defense response, incompatible interaction	6	5.30E-05	P
GO:1901141	regulation of lignin biosynthetic process	2	0.00049	P
GO:0009809	lignin biosynthetic process	3	0.00071	P
GO:0071446	cellular response to salicylic acid stimulus	3	8.00E-04	P
GO:0044710	single-organism metabolic process	42	0.00085	P
GO:0020037	heme binding	22	5.40E-10	F
GO:0046906	tetrapyrrole binding	22	6.90E-10	F
GO:0010333	terpene synthase activity	7	1.10E-06	F
GO:0016838	carbon-oxygen lyase activity, acting on phosphates	7	1.50E-06	F
GO:0016491	oxidoreductase activity	31	1.70E-06	F
GO:0016705	oxidoreductase activity, acting on paired donors, with incorporation or reduction of molecular oxygen	15	5.20E-06	F
GO:0005506	iron ion binding	15	6.00E-06	F
GO:0016835	carbon-oxygen lyase activity	8	1.20E-05	F
GO:0004601	peroxidase activity	8	2.70E-05	F
GO:0016684	oxidoreductase activity, acting on peroxide as acceptor	8	3.20E-05	F
GO:0016829	lyase activity	10	4.40E-05	F
GO:0016209	antioxidant activity	8	6.70E-05	F
GO:0003824	catalytic activity	78	7.20E-05	F
GO:0000287	magnesium ion binding	7	7.40E-05	F
P. inflata DEGs down-regulated by PhERF1				
GO:0055114	oxidation-reduction process	32	2.00E-06	P
GO:0002215	defense response to nematode	2	0.00013	P
GO:0006979	response to oxidative stress	10	0.00027	P
GO:0044710	single-organism metabolic process	44	0.00035	P
GO:0020037	heme binding	24	6.50E-12	F
GO:0046906	tetrapyrrole binding	24	9.20E-12	F
GO:0016491	oxidoreductase activity	33	5.10E-08	F
GO:0004601	peroxidase activity	10	2.70E-07	F
GO:0016684	oxidoreductase activity, acting on peroxide as acceptor	10	3.20E-07	F
GO:0016209	antioxidant activity	10	7.50E-07	F
GO:0016705	oxidoreductase activity, acting on paired donors, with incorporation or reduction of molecular oxygen	15	2.40E-06	F
GO:0005506	iron ion binding	15	3.50E-06	F
GO:0003824	catalytic activity	78	3.90E-06	F
GO:0010333	terpene synthase activity	6	6.80E-06	F
GO:0016835	carbon-oxygen lyase activity	8	7.40E-06	F
GO:0016838	carbon-oxygen lyase activity, acting on phosphates	6	8.90E-06	F
GO:0016829	lyase activity	9	0.00015	F
GO:0000287	magnesium ion binding	6	0.00048	F

Note: P; biological process, F; molecular function, C; cellular component.

Table S5 Tomato proteins involved in sterol biosynthesis

Abbreviation	Gene ID	Description
ACAT	Solyc05g017760	acetyl-CoA C-acetyltransferase
HMGS	Solyc08g080170	hydroxymethylglutaryl-CoA synthase
HMGR	Solyc02g082260	hydroxymethylglutaryl-CoA reductase
MVK	Solyc01g098840	mevalonate kinase
PMK	Solyc06g066310	phosphomevalonate kinase
MPPDC	Solyc04g009650	mevalonate diphosphate decarboxylase
IDI	Solyc04g056390	isopentenyl diphosphate isomerase
FPPS	Solyc12g015860	farnesyl diphosphate synthase
SQS	Solyc01g110290	squalene synthase
SQO	Solyc04g077440	squalene monooxygenase
CAS	Solyc04g070980	cycloartenol synthase
SSR2	Solyc02g069490	sterol side chain reductase 2
SMO3	Solyc01g091320	sterol methyl oxidase 3
SMO4	Solyc06g005750	sterol methyl oxidase 4
DWF7-2	Solyc02g086180	sterol C-5 desaturase / Dwarf 7-2
DWF5-2	Solyc06g074090	sterol reductase / Dwarf 5-2
ERG28	Solyc04g077150	ERG28-like protein
HSD	Solyc02g032330	3β-hydroxysteroid dehydrogenase
CPI	Solyc12g098640	cyclopropylsterol isomerase
CYP51G1	Solyc01g008110	sterol C14-demethylase / CYP51G
HYD2	Solyc09g009040	Δ14-sterol reductase
HYD1	Solyc06g082980	3β-hydroxysteroid-Δ8Δ7-isomerase
SMT1	Solyc10g080150	sterol methyltransferase 1
SMO1	Solyc08g079570	sterol methyl oxidase 1
SMO2	Solyc06g076410	sterol methyl oxidase 2
DWF7-1	Solyc02g063240	sterol C-5 desaturase / Dwarf 7-1
DWF5-1	Solyc01g009310	sterol reductase / Dwarf 5-1
SMT2	Solyc01g111830	sterol methyltransferase 1
SSR1	Solyc02g030170	sterol side chain reductase 1
CYP710A11	Solyc02g070580	sterol 22-desaturase / CYP710A

Table S6 Petunia genes homologous to tomato sterol biosynthesis genes

gene name	gene ID	description
PhACAT1	Peaxi162Scf00802g00025	acetyl-CoA C-acetyltransferase
PhACAT2	Peaxi162Scf00811g00219	acetyl-CoA C-acetyltransferase
PhACAT3	Peinf101Scf00265g03022	acetyl-CoA C-acetyltransferase
PhACAT4	Peinf101Scf01292g02028	acetyl-CoA C-acetyltransferase
PhHMGS1	Peaxi162Scf00102g01349	hydroxymethylglutaryl-CoA synthase
PhHMGS2	Peaxi162Scf00620g00815	hydroxymethylglutaryl-CoA synthase
PhHMGS3	Peaxi162Scf00620g00831	hydroxymethylglutaryl-CoA synthase
PhHMGS4	Peaxi162Scf00620g00832	hydroxymethylglutaryl-CoA synthase
PhHMGS5	Peaxi162Scf01294g00241	hydroxymethylglutaryl-CoA synthase
PhHMGS6	Peinf101Scf00340g00011	hydroxymethylglutaryl-CoA synthase
PhHMGS7	Peinf101Scf00340g00012	hydroxymethylglutaryl-CoA synthase
PhHMGS8	Peinf101Scf00340g00018	hydroxymethylglutaryl-CoA synthase
PhHMGS9	Peinf101Scf02382g06025	hydroxymethylglutaryl-CoA synthase
PhHMGS10	Peinf101Scf03855g00043	hydroxymethylglutaryl-CoA synthase
PhHMGS11	Peinf101Scf05410g01043	hydroxymethylglutaryl-CoA synthase
PhHMGR1	Peaxi162Scf00385g00039	hydroxymethylglutaryl-CoA reductase
PhHMGR2	Peaxi162Scf00431g00310	hydroxymethylglutaryl-CoA reductase
PhHMGR3	Peaxi162Scf00861g00321	hydroxymethylglutaryl-CoA reductase
PhHMGR4	Peaxi162Scf01393g00015	hydroxymethylglutaryl-CoA reductase
PhHMGR5	Peinf101Scf00393g02016	hydroxymethylglutaryl-CoA reductase
PhHMGR6	Peinf101Scf00409g05014	hydroxymethylglutaryl-CoA reductase
PhHMGR7	Peinf101Scf00774g02014	hydroxymethylglutaryl-CoA reductase
PhHMGR8	Peinf101Scf00982g06021	hydroxymethylglutaryl-CoA reductase
PhMVK1	Peaxi162Scf00037g00226	mevalonate kinase
PhMVK2	Peaxi162Scf00471g00018	mevalonate kinase
PhMVK3	Peinf101Scf00511g09010	mevalonate kinase
PhMVK4	Peinf101Scf01272g04039	mevalonate kinase
PhPMK1	Peaxi162Scf00052g00324	phosphomevalonate kinase
PhPMK2	Peaxi162Scf00185g01121	phosphomevalonate kinase
PhPMK3	Peinf101Scf00536g14013	phosphomevalonate kinase
PhPMK4	Peinf101Scf02167g00007	phosphomevalonate kinase
PhMPPDC1	Peaxi162Scf00173g00046	mevalonate diphosphate decarboxylase
PhMPPDC2	Peaxi162Scf00519g00710	mevalonate diphosphate decarboxylase
PhMPPDC3	Peinf101Scf00445g00015	mevalonate diphosphate decarboxylase
PhMPPDC4	Peinf101Scf01790g01038	mevalonate diphosphate decarboxylase
PhIDI1	Peaxi162Scf00048g00815	isopentenyl diphosphate isomerase
PhIDI2	Peaxi162Scf00156g00114	isopentenyl diphosphate isomerase
PhIDI3	Peinf101Scf00519g05011	isopentenyl diphosphate isomerase
PhIDI4	Peinf101Scf01201g03020	isopentenyl diphosphate isomerase
PhFPPS1	Peaxi162Scf00006g00522	farnesyl diphosphate synthase
PhFPPS2	Peinf101Scf00071g09019	farnesyl diphosphate synthase
PhSQS1	Peaxi162Scf00003g00613	squalene synthase
PhSQS2	Peaxi162Scf00003g04242	squalene synthase
PhSQS3	Peaxi162Scf00016g00152	squalene synthase
PhSQS4	Peaxi162Scf00285g00432	squalene synthase
PhSQS5	Peinf101Scf00019g17029	squalene synthase
PhSQS6	Peinf101Scf00835g00020	squalene synthase
PhSQS7	Peinf101Scf02318g05033	squalene synthase
PhSQO1	Peaxi162Scf00152g00612	squalene monooxygenase
PhSQO2	Peaxi162Scf00449g00512	squalene monooxygenase
PhSQO3	Peaxi162Scf01161g00325	squalene monooxygenase
PhSQO4	Peinf101Scf00395g05007	squalene monooxygenase
PhSQO5	Peinf101Scf00650g29067	squalene monooxygenase
PhSQO6	Peinf101Scf00736g22027	squalene monooxygenase
PhSQO7	Peinf101Scf00932g02019	squalene monooxygenase
PhCAS1	Peaxi162Scf00263g00924	cycloartenol synthase
PhCAS2	Peaxi162Scf00443g00213	cycloartenol synthase
PhCAS3	Peaxi162Scf00928g00242	cycloartenol synthase
PhCAS4	Peaxi162Scf00954g00115	cycloartenol synthase
PhCAS5	Peaxi162Scf00984g00023	cycloartenol synthase
PhCAS6	Peinf101Scf00172g00004	cycloartenol synthase
PhCAS7	Peinf101Scf00296g04013	cycloartenol synthase
PhCAS8	Peinf101Scf00413g11025	cycloartenol synthase
PhCAS9	Peinf101Scf00810g00010	cycloartenol synthase
PhCAS10	Peinf101Scf00978g04022	cycloartenol synthase
PhCAS11	Peinf101Scf02137g00058	cycloartenol synthase
PhCAS12	Peinf101Scf02326g03026	cycloartenol synthase
PhERG28.1	Peaxi162Scf00628g00517	ERG28-like protein
PhERG28.2	Peinf101Scf00736g11022	ERG28-like protein
PhHSD1	Peaxi162Scf00198g01529	3β-hydroxysteroid dehydrogenase
PhHSD2	Peaxi162Scf00368g00814	3β-hydroxysteroid dehydrogenase
PhHSD3	Peinf101Scf00737g02029	3β-hydroxysteroid dehydrogenase
PhHSD4	Peinf101Scf01538g02028	3β-hydroxysteroid dehydrogenase
PhCPI1	Peaxi162Scf00378g00519	cyclopropylsterol isomerase
PhCPI2	Peinf101Scf00526g04007	cyclopropylsterol isomerase
PhCYP51G.1	Peaxi162Scf00097g00139	sterol C14-demethylase / CYP51G
PhCYP51G.2	Peinf101Ctg13765786g0000	sterol C14-demethylase / CYP51G
PhCYP51G.3	Peinf101Scf00763g05002	sterol C14-demethylase / CYP51G

gene name	gene ID	description
PhACAT1	Peaxi162Scf00802g00025	acetyl-CoA C-acetyltransferase
PhACAT2	Peaxi162Scf00811g00219	acetyl-CoA C-acetyltransferase
PhACAT3	Peinf101Scf00265g03022	acetyl-CoA C-acetyltransferase
PhACAT4	Peinf101Scf01292g02028	acetyl-CoA C-acetyltransferase
PhHMGS1	Peaxi162Scf00102g01349	hydroxymethylglutaryl-CoA synthase
PhHMGS2	Peaxi162Scf00620g00815	hydroxymethylglutaryl-CoA synthase
PhHMGS3	Peaxi162Scf00620g00831	hydroxymethylglutaryl-CoA synthase
PhHMGS4	Peaxi162Scf00620g00832	hydroxymethylglutaryl-CoA synthase
PhHMGS5	Peaxi162Scf01294g00241	hydroxymethylglutaryl-CoA synthase
PhHMGS6	Peinf101Scf00340g00011	hydroxymethylglutaryl-CoA synthase
PhHMGS7	Peinf101Scf00340g00012	hydroxymethylglutaryl-CoA synthase
PhHMGS8	Peinf101Scf00340g00018	hydroxymethylglutaryl-CoA synthase
PhHMGS9	Peinf101Scf02382g06025	hydroxymethylglutaryl-CoA synthase
PhHMGS10	Peinf101Scf03855g00043	hydroxymethylglutaryl-CoA synthase
PhHMGS11	Peinf101Scf05410g01043	hydroxymethylglutaryl-CoA synthase
PhHMGR1	Peaxi162Scf00385g00039	hydroxymethylglutaryl-CoA reductase
PhHMGR2	Peaxi162Scf00431g00310	hydroxymethylglutaryl-CoA reductase
PhHMGR3	Peaxi162Scf00861g00321	hydroxymethylglutaryl-CoA reductase
PhHMGR4	Peaxi162Scf01393g00015	hydroxymethylglutaryl-CoA reductase
PhHMGR5	Peinf101Scf00393g02016	hydroxymethylglutaryl-CoA reductase
PhHMGR6	Peinf101Scf00409g05014	hydroxymethylglutaryl-CoA reductase
PhHMGR7	Peinf101Scf00774g02014	hydroxymethylglutaryl-CoA reductase
PhHMGR8	Peinf101Scf00982g06021	hydroxymethylglutaryl-CoA reductase
PhMVK1	Peaxi162Scf00037g00226	mevalonate kinase
PhMVK2	Peaxi162Scf00471g00018	mevalonate kinase
PhMVK3	Peinf101Scf00511g09010	mevalonate kinase
PhMVK4	Peinf101Scf01272g04039	mevalonate kinase
PhPMK1	Peaxi162Scf00052g00324	phosphomevalonate kinase
PhPMK2	Peaxi162Scf00185g01121	phosphomevalonate kinase
PhPMK3	Peinf101Scf00536g14013	phosphomevalonate kinase
PhPMK4	Peinf101Scf02167g00007	phosphomevalonate kinase
PhMPPDC1	Peaxi162Scf00173g00046	mevalonate diphosphate decarboxylase
PhMPPDC2	Peaxi162Scf00519g00710	mevalonate diphosphate decarboxylase
PhMPPDC3	Peinf101Scf00445g00015	mevalonate diphosphate decarboxylase
PhMPPDC4	Peinf101Scf01790g01038	mevalonate diphosphate decarboxylase
PhIDI1	Peaxi162Scf00048g00815	isopentenyl diphosphate isomerase
PhIDI2	Peaxi162Scf00156g00114	isopentenyl diphosphate isomerase
PhIDI3	Peinf101Scf00519g05011	isopentenyl diphosphate isomerase
PhIDI4	Peinf101Scf01201g03020	isopentenyl diphosphate isomerase
PhFPPS1	Peaxi162Scf00006g00522	farnesyl diphosphate synthase
PhFPPS2	Peinf101Scf00071g09019	farnesyl diphosphate synthase
PhSQS1	Peaxi162Scf00003g00613	squalene synthase
PhSQS2	Peaxi162Scf00003g04242	squalene synthase
PhSQS3	Peaxi162Scf00016g00152	squalene synthase
PhSQS4	Peaxi162Scf00285g00432	squalene synthase
PhSQS5	Peinf101Scf00019g17029	squalene synthase
PhSQS6	Peinf101Scf00835g00020	squalene synthase
PhSQS7	Peinf101Scf02318g05033	squalene synthase
PhSQO1	Peaxi162Scf00152g00612	squalene monooxygenase
PhSQO2	Peaxi162Scf00449g00512	squalene monooxygenase
PhSQO3	Peaxi162Scf01161g00325	squalene monooxygenase
PhSQO4	Peinf101Scf00395g05007	squalene monooxygenase
PhSQO5	Peinf101Scf00650g29067	squalene monooxygenase
PhSQO6	Peinf101Scf00736g22027	squalene monooxygenase
PhSQO7	Peinf101Scf00932g02019	squalene monooxygenase
PhCAS1	Peaxi162Scf00263g00924	cycloartenol synthase
PhCAS2	Peaxi162Scf00443g00213	cycloartenol synthase
PhCAS3	Peaxi162Scf00928g00242	cycloartenol synthase
PhCAS4	Peaxi162Scf00954g00115	cycloartenol synthase
PhCAS5	Peaxi162Scf00984g00023	cycloartenol synthase
PhCAS6	Peinf101Scf00172g00004	cycloartenol synthase
PhCAS7	Peinf101Scf00296g04013	cycloartenol synthase
PhCAS8	Peinf101Scf00413g11025	cycloartenol synthase
PhCAS9	Peinf101Scf00810g00010	cycloartenol synthase
PhCAS10	Peinf101Scf00978g04022	cycloartenol synthase
PhCAS11	Peinf101Scf02137g00058	cycloartenol synthase
PhCAS12	Peinf101Scf02326g03026	cycloartenol synthase
PhERG28.1	Peaxi162Scf00628g00517	ERG28-like protein
PhERG28.2	Peinf101Scf00736g11022	ERG28-like protein
PhHSD1	Peaxi162Scf00198g01529	3β-hydroxysteroid dehydrogenase
PhHSD2	Peaxi162Scf00368g00814	3β-hydroxysteroid dehydrogenase
PhHSD3	Peinf101Scf00737g02029	3β-hydroxysteroid dehydrogenase
PhHSD4	Peinf101Scf01538g02028	3β-hydroxysteroid dehydrogenase
PhCPI1	Peaxi162Scf00378g00519	cyclopropylsterol isomerase
PhCPI2	Peinf101Scf00526g04007	cyclopropylsterol isomerase
PhCYP51G.1	Peaxi162Scf00097g00139	sterol C14-demethylase / CYP51G
PhCYP51G.2	Peinf101Ctg13765786g0000	sterol C14-demethylase / CYP51G
PhCYP51G.3	Peinf101Scf00763g05002	sterol C14-demethylase / CYP51G

Table S7 Summary of GC-MS analysis targeting sterols

	peak area							
metabolites	control_1	control_2	control_3	control_4	control_5	control_6	control_7	control_8
5α-cholestane (internal standard)	227,882	227,180	238,857	217,815	219,834	219,719	217,252	220,988
cholesterol	15,628	11,032	14,299	12,917	16,166	15,828	29,004	8,662
24-methylenecholesterol	16,692	14,534	28,761	24,116	22,531	23,278	15,959	14,422
campesterol	121,358	94,085	143,907	109,839	125,809	113,981	113,042	85,147
stigmasterol	48,258	32,066	50,564	35,269	46,322	35,766	34,471	32,093
24-methyldesmosterol	83,178	85,253	178,651	158,886	122,083	119,457	99,677	88,922
β-sitosterol	151,001	108,757	123,203	96,538	122,833	109,187	107,176	79,636

	peak area							
metabolites	PhERF1 OX_1	PhERF1 OX_2	PhERF1 OX_3	PhERF1 OX_4	PhERF1 OX_5	PhERF1 OX_6	PhERF1 OX_7	PhERF1 OX_8
5α-cholestane (internal standard)	221,464	222,791	205,864	215,752	204,075	213,821	210,452	212,956
cholesterol	11,798	11,242	10,309	11,976	13,028	10,697	9,167	8,507
24-methylenecholesterol	23,210	24,612	21,629	24,430	31,402	27,958	22,780	17,577
campesterol	129,960	174,593	131,751	158,410	191,387	186,437	153,292	101,288
stigmasterol	28,843	42,159	35,270	34,578	39,658	41,104	38,007	25,094
24-methyldesmosterol	56,158	60,981	53,330	53,260	79,385	86,281	57,370	60,408
β-sitosterol	75,284	117,266	118,657	121,899	121,091	122,382	106,288	118,534

	peak area									
metabolites	PhERF1 OX_9	PhERF1 OX_10	PhERF1 OX_11	PhERF1 OX_12	PhERF1 OX_13	PhERF1 OX_14	PhERF1 OX_15	PhERF1 OX_16	PhERF1 OX_17	PhERF1 OX_18
5α-cholestane (internal standard)	217,395	198,520	198,807	203,935	207,871	208,040	199,884	196,710	194,644	190,163
cholesterol	11,616	11,388	10,782	10,060	11,998	12,289	9,696	9,391	10,132	8,427
24-methylenecholesterol	17,773	22,110	13,337	17,474	26,143	25,738	25,788	21,215	20,462	22,017
campesterol	136,174	171,131	117,065	148,782	153,931	171,990	153,131	144,722	130,981	163,047
stigmasterol	35,429	38,361	31,107	33,815	33,913	39,967	30,685	35,690	27,385	37,503
24-methyldesmosterol	43,099	50,146	32,033	44,206	68,301	65,191	61,031	52,077	65,481	57,596
β-sitosterol	114,798	97,624	88,437	93,192	97,111	132,589	87,507	110,316	84,967	128,728

Table S8 Oligonucleotide primers used in RT-qPCR analysis

Gene name	Gene ID or accession No.	forward/reverse (F/R)	sequence (5' to 3')
PhERF1	Peaxi162Scf00672g00031	F	ATGACCAAGCTGCTTTCAAGTTTCG
		R	CCTCGAAGTCAGCCTAGCCGGATTAGGC
PhERF7	Peaxi162Scf00475g00053	F	GAGAAAGCAGCTGGGGAGATATGGTTCC
		R	CACAGGCTCCTCCTTTACTTCCTTCAGCTCC
PhERF10	Peaxi162Scf00199g00056	F	ATGCAGCTTGGTCATATTTCATCAG
		R	GAGTTGCTGCTAGGGGTTTGGAATATTGAG
PhCAS1	Peaxi162Sch00263g00924	F	GTGAAATGAGTGGCATTTCTGG
		R	TAGCCAGTTAAAGGTTGACAGG
PhSMO1.2	Peinf101Scf00284g004025	F	CATAGATCTTCAGAGGTCTTATGA
		R	CTCTAATCTTATCTACTGCCTTGACAC
Ph24ISO2	Peaxi162Scf00695g00218	F	CAGATGCAAAGGCCCCTGTTCG
		R	TAGGCACGAGAAAGGAAGGAC
PhCYP88C13.1	Peaxi162Scf00312g00220	F	CTGGATATAATGCTAAGCACTC
		R	CTTAGCAACAGATTCATAACC
GUS	AF485783	F	CTTTGATCGCGTCAGCGCCGTCG
		R	CGAAGTTCATGCCAGTCCAGCG
EF1a	Peaxi162Scf00351g00312	F	AAGCCTGGTATGGTTGTGAC
		R	GAACCCAACATTGTCACCAGG

Dataset S1 (separate file). Expression dataset of *P. axillaris* genes in the *PhERF1* overexpression experiment.

Dataset S2 (separate file). Expression dataset of *P. inflata* genes in the *PhERF1* overexpression experiment.

Dataset S3 (separate file). Expression dataset of *P. axillaris* genes in the MeJA treatment experiment.

Dataset S4 (separate file). Expression dataset of *P. inflata* genes in the MeJA treatment experiment.

Dataset S5 (separate file). Summary of LC-MS analysis. Cells are colored according to peak area values, with red representing the maximum value and blue representing the minimum value.

Dataset S6 (separate file). Select peaks of LC-MS analysis. Cells are colored according to peak area values, with red representing the maximum value and blue representing the minimum value.

Dataset S7 (separate file). Summary of comprehensive GC-MS analysis. Cells are colored according to peak area values, with red representing the maximum value and blue representing the minimum value.
